# Age-dependent associations of human milk oligosaccharides with body size and composition up to 4 years of age

**DOI:** 10.1016/j.ajcnut.2023.02.016

**Published:** 2023-02-21

**Authors:** Toby Mansell, Annalee Furst, Martin O’Hely, Melinda Chang, Anne-Louise Ponsonby, Peter Vuillermin, Mimi LK. Tang, David Burgner, Richard Saffery, Lars Bode, Barwon Infant Study Investigator Group

**Affiliations:** 1 Murdoch Children’s Research Institute, Royal Children’s Hospital, Parkville, Australia; 2 Department of Paediatrics, University of Melbourne, Parkville, Australia; 3 Department of Pediatrics and Mother-Milk-Infant Center of Research Excellence, University of California, San Diego, California, United States; 4 School of Medicine, Deakin University, Geelong, Australia; 5 The Florey Institute of Neuroscience and Mental Health, Parkville, Australia; 6 Child Health Research Unit, Barwon Health, Geelong, Australia; 7 Department of Paediatrics, Monash University, Clayton, Australia

**Keywords:** human milk oligosaccharides, secretor, growth, anthropometry, adiposity, 2’FL

## Abstract

**Background:**

Human milk oligosaccharides (HMOs) are major components of human milk that may mediate its beneficial effects on infant growth.

**Objectives:**

To investigate relationships between HMO concentrations in milk at 6 wk postpartum and anthropometry to 4 y of age in human milk-fed infants.

**Methods:**

Milk samples were collected from 292 mothers at 6 wk (median 6.0 wk; range 3.3, 11.1] postpartum in a longitudinal, population-derived cohort. Of the infants, 171 were exclusively human milk-fed to 3 mo of age and 127 to 6 mo. Concentrations of 19 HMOs were quantified using high-performance liquid chromatography. Maternal secretor status (*n* = 221 secretors) was determined from 2’-fucosyllactose (2’FL) concentration. We calculated *z*-scores for child weight, length, head circumference, summed triceps and subscapular skinfold thicknesses, and weight-for-length at 6 wk, 6 mo, 12 mo, and 4 y. We investigated associations of secretor status and each HMO measure with change from birth for each *z*-score using linear mixed-effects models.

**Results:**

Maternal secretor status was not associated with anthropometric *z*-scores up to 4 y of age. Several HMOs were associated with *z*-scores at 6 wk and 6 mo, predominantly within secretor status subgroups. Higher levels of 2’FL were associated with greater weight [β = 0.91 increase in *z*-score per SD increase log-2’FL, 95% CI (0.17, 1.65)] and length [β = 1.22, (0.25, 2.20)] in children born to secretor mothers, but not body composition measures. Higher lacto-N-tetraose was associated with greater weight [β = 0.22, (0.02, 0.41)] and length (β = 0.30, (0.07, 0.53)] among children born to nonsecretor mothers. Several HMOs were associated with anthropometric measures at 12 mo and 4 y of age.

**Conclusions:**

Milk HMO composition at 6 wk postpartum is associated with several anthropometry measures up to 6 mo of age in a potential secretor status-specific manner, with largely different HMOs associating with anthropometry from 12 mo to 4 y of age.

## Introduction

Human milk oligosaccharides (HMOs) are complex carbohydrates of diverse structures [1] that represent the fourth most abundant components in human milk after water, lactose, and lipids [2]. The diversity of HMOs in human milk is 1 of the major differences in composition relative to other animals and formulas.

Accumulating evidence suggests that HMOs have many direct and indirect effects on infant health. HMOs are prebiotics that enrich the colonization of beneficial bacteria in the infant gastrointestinal microbiome [3] and modify the circulating SCFA profile [4], including increasing acetate [5]. HMOs also modulate cellular immune responses [6,7] and reduce the risk of infection by binding to pathogens and preventing adherence to epithelial cells [8,9]. They also have direct antibacterial [10] and antifungal [11] activity. The effects of HMOs on the gastrointestinal microbiome and immune function may also influence a range of infant health outcomes, including promoting healthy early growth and protection against obesity.

Recent studies have reported associations between HMO concentrations and early childhood weight and length [12,13] and fat mass [14], with the strongest relationship reported for 2’-fucosyllactose (2’FL) (positively associated with weight and length) and lacto-N-neotetraose (LNnT) (negatively associated), specific to children born to “secretor” mothers, with high levels of milk 2’FL. However, as early-life growth trajectory influences the later risk of obesity [15] and the emergence of other metabolic risk factors [16] in childhood, these relationships require a deeper understanding, particularly those regarding persistent effects on growth after human milk feeding ceases, as suggested by previous studies, with evidence for effects on height up to 5 y of age [12]. In addition, although length, weight, and fat mass have been investigated in previous studies, it is currently unknown how HMO concentrations relate to other early-life anthropometric measures that are predictive of later health outcomes, such as a sum of triceps and subscapular skinfold thicknesses (a measure of central adiposity) or head circumference.

This study investigated the relationship between HMO milk concentrations at 6 wk postpartum and early-life growth (change from birth) in body composition measures (hereafter anthropometry), both during and after lactation. In addition to investigating the early-life sum of skinfold thicknesses and head circumference for the first time, we sought to replicate previous findings from cohorts in the United States [14], Denmark [13], and Finland [12] and investigate the evidence for these associations persisting up to 4 y of age.

## Methods

### Study cohort

Data were used from all mother-child dyads with available 6-wk postpartum milk samples, child anthropometric measurements from at least 1-time point, and complete covariate data (*n* = 292, 1 twin pair) in the Barwon Infant Study, a longitudinal, prebirth cohort in the south-east of Australia (*n* = 1064 pregnancies)[17]. Mothers were recruited during their antenatal visit to local hospitals at approximately 15 wk gestation and provided informed written consent. The inclusion criteria for the Barwon Infant Study were that mothers were residents of the Barwon region and intended to give birth at the local public or private hospital. Exclusion criteria were mothers who were not permanent Australian residents, mothers <18 y of age, those requiring an interpreter to complete questionnaires, moving out of the Barwon region prior to birth, those planning to store their child’s cord blood privately, or those who participated previously in the study. Child exclusion criteria were gestational age of <32 completed weeks or diagnosis of a serious illness or congenital disease within the first few days of life. A flowchart of participant inclusion is shown in Supplementary Figure 1. Ethics approval was granted by the Barwon Health Human Research ethics committee (HREC 10/24). The procedures followed were in accordance with the Helsinki Declaration of 1975, as revised in 1983.

### Milk collection and HMO quantification

Milk (foremilk) was collected from mothers during the 6-wk time point visit (median 6.0 wk; range 3.3, 11.1) at least 2 h after the last infant feed. Visits occurred throughout the day. Mothers were given the option of hand express or a provided pump, and a volume of 10–20 mL was expressed into sterile containers. Milk samples were immediately refrigerated and brought to the research center on ice blocks. All samples were aliquoted and stored at −80°C within 24 h of collection. Frozen aliquots (1.7 mL) were shipped to the University of California, San Diego, CA, on dry ice for HMO quantification as previously described [18]. In brief, concentrations of 19 HMOs were measured by high-performance liquid chromatography on an amide-80 column with fluorescent detection, using the oligosaccharide raffinose as an internal standard. HMOs were: 2’FL, 3-fucosyllactose (3FL), 3’-sialyllactose (3’SL), 6’-sialyllactose (6’SL), difucosyllactose, difucosyllacto-N-hexaose, difucosyllacto-N-tetraose, disialyllacto-N-hexaose (DSLNH), disialyllacto-N-tetraose (DSLNT), fucodisialyllacto-N-hexaose (FDSLNH), fucosyllacto-N-hexaose, lacto-N-fucopentaose (LNFP) I, LNFP II, LNFP III, lacto-N-hexaose (LNH), LNnT, lacto-N-tetraose (LNT), sialyl-lacto-N-tetraose b (LSTb), and sialyl-lacto-N-tetraose c. Total HMO concentration was calculated as the sum of the concentrations of the 19 measured oligosaccharides. As there was previous evidence for associations between the ratio of 2’FL and LNnT (2’FL/LNnT) and infant growth [12], this ratio was considered an HMO measure. HMO-bound fucose (fuc) and HMO-bound sialic acid (sia) were calculated on a molar basis. The proportion of each HMO comprising the total HMO concentration was also calculated. HMO Simpson’s diversity index was calculated [19] based on the relative abundances of all 19 HMOs. The higher the diversity value, the more heterogenous the HMO composition in the sample. Maternal secretor status was determined based on the presence (secretor) or near absence (<100 nmol/mL) of 2’FL (nonsecretor).

### Child anthropometric measures

Five anthropometric measures were *z*-scores derived for weight, length (in infants) or height (in childhood), weight-for-length, head circumference, and the sum of triceps and subscapular skinfold thickness at the birth, 6-wk, 6-mo, 12-mo, and 4-y time points. The exact age at each time point was used for *z*-score calculations. Growth was considered to be the change from birth *z*-score. Measurements of weight and length/height (referred to as length hereafter) were taken in light clothes and without shoes. Length measurements were taken from hospital birth records for birth length, measured by measuring mat (Seca GmbH; Seca mobile measuring mat 210) for infants up to 12 mo of age, and by stadiometer (Seca 213 Portable Height Measuring Rod Stadiometer) for children >12 mo of age. Length measures were made in duplicate, with a third measurement made if the first 2 differed by more than 1.0 cm. The mean of the replicate measurements, rounded to the nearest 0.5 cm up to 12 mo and 0.1 cm at 4 y, was used for analysis. Weight measurements were taken from hospital birth records for birth weight and measured by digital scale for all other time points (Seca Digital Baby Scale 354 for infants up to 12 mo of age; Omron: Omron Digital Weight Scale Model: HN-286 for children >12 mo). Weight measures were made in duplicate, with a third measurement made if the first 2 differed by more than 0.3 kg. The mean of the replicate measurements, to 2 decimal places, was used for analysis. Head circumference measurements were taken from hospital birth records for birth head circumference and measured by measuring tape (Seca measuring tape 212) at other time points. Measurements were made in duplicate, with a third measurement made if the first 2 differed by more than 0.3 cm. The mean of the replicate measures rounded to the nearest 0.25 cm was used for analysis. Triceps and subscapular skinfold thickness were measured using calipers (Holtain: Holtain Skinfold Calipers) [20]. Measurements were made in duplicate, with a third measurement made if the first 2 differed by more than 2.0 mm. The mean of replicate measures for triceps and subscapular skinfold thicknesses were summed, and the sum of skinfold thickness was used in analyses. Age-and-sex standardized *z*-scores for weight, length, and weight-for-length were based on the WHO growth standards [21]. As head circumference and the sum of skinfold thickness *z*-scores are not included in these growth standards, cohort-specific *z*-scores were calculated by standardizing head circumference and the sum of skinfold thickness by sex at each time point.

### Maternal and child covariates

Covariates for models were maternal prepregnancy BMI (in kg/m^2^) (calculated from self-reported weight and height), which is associated with differences in HMO concentrations [22], household income during pregnancy (self-reported), child sex, and duration of human milk feeding and postnatal age at introduction of formula milk feeding (both reported in questionnaires). The week of the introduction of formula milk was characterized by 2 dichotomous variables: any formula feeding by 6 mo of age and any formula feeding by 12 mo for main analyses. Introduction to any solid food by 3, 6, 9, and 12 mo of age was considered as a covariate in secondary analyses.

### Statistical analysis

Analyses were performed in R version 4.0.2 [23]. Distribution of cohort characteristics and milk HMO concentrations are reported as median and IQR. Prior to analysis, HMO concentrations and 2’FL/LNnT ratio were log-transformed. All HMO measures were scaled to a standardized distribution (SD units) to allow for visual comparison across HMOs. Principal component analysis was used to visualize variation in HMO composition (‘ggbiplot’ package version 0.55).

Two sets of hierarchical linear mixed-effects models were used (‘nlme’ package version 3.1-152), with separate models for the 120 combinations of the 24 HMO measures (refer to Supplementary Table 1) as the exposure and the 5 anthropometric *z*-scores as the outcome. Child identifier (which measurements at each time point were nested within) was included as a random effect, and all other covariates were included as fixed effects. The first models considered anthropometric *z*-scores at 6 wk and 6 mo of age as the outcome, as the majority (75.2%) of children were human milk-fed until at least 6 mo of age. The second set considered anthropometric *z*-scores at 12 mo and 4 y of age to investigate persistent associations of milk HMO concentration with growth. All models were adjusted for maternal prepregnancy BMI, household income during pregnancy, child sex, human milk feeding duration in completed weeks (up to 6 mo or up to 12 mo, respectively), the introduction of formula milk feeding by 6 mo or 12 mo of age (respectively) and the corresponding *z*-score at birth. All models included an interaction term between HMO and time point and used an unstructured correlation structure and a random intercept for each participant. In secondary analyses, we considered models additionally adjusted for the introduction of solid food timing and models adjusted for the week of formula feeding introduction instead of the categorical formula feeding variables.

As the milk composition of many of the measured HMOs differs substantially by the secretor status of mothers [24,25], all models were tested in both the whole cohort and stratified by secretor status. In addition, sex-stratified models with and without secretor status-stratification were also investigated to descriptively consider differences in associations of HMO concentration and secretor status with child anthropometry by sex.

Post hoc investigation of model assumptions for all main findings was performed by visually assessing model residuals for linearity, normality, and homoscedasticity. In addition, the homogeneity of residual variance across participants was assessed with Levene’s test [26].

## Results

### Cohort characteristics

Of the 292 mother-child dyads in this study, 221 (75.7%) mothers were classified as secretors (>100 nmol/mL of 2’FL) and 71 (24.3%) as nonsecretors (<100 nmol/mL). The cohort characteristics, including secretor status, are shown in Table 1. Distribution of HMO concentrations at 6 wk postpartum is shown in Supplementary Table 1, with higher α1-2 fucosylated and total HMOs in the secretor group and higher concentrations of LNFP II and FDSLNH in the nonsecretor group. Secretor status appeared to be a major determinant of HMO composition, explaining the largest principal component of variation of all HMO data (31.9% variation) (Supplementary Figure 2). Similar patterns were observed for variation in HMO data excluding 2’FL (as 2’FL was used to determine secretor status) (Supplementary Figure 3).

**TABLE 1:** Summary of cohort characteristics, by secretor status

Measure	*n*	Combined	Nonsecretor	Secretor
		*n* = 292	*n* = 71	*n* = 221
Sex (male)	292	155 (53%)	35 (49%)	120 (54%)
Maternal prepregnancy BMI (kg/m^2^)	292	23.7 (21.4–27.5)	24.2 (21.6–27.1)	23.5 (21.3–27.5)
Household income (AUD)	292			
>25,000		3 (1.0%)	1 (1.4%)	2 (0.9%)
25,000–49,999		15 (5.1%)	3 (4.2%)	12 (5.4%)
50,000–74,999		38 (13%)	7 (9.9%)	31 (14%)
75,000–99,999		78 (27%)	17 (24%)	61 (28%)
100,000–149,999		111 (38%)	34 (48%)	77 (35%)
≥150,000		47 (16%)	9 (13%)	38 (17%)
Duration of any human milk feeding (maximum 52 wk)	292	50 (24–52)	46 (23–52)	51 (24–52)
Introduction to formula feeding (any)	292			
By 6 mo of age		163 (55.8%)	43 (60.5%)	120 (54.3%)
By 12 mo of age		199 (68.2%)	51 (71.8%)	148 (70.0%)
**Birth**				
Gestational age (wk)	292	39.6 (39.0–40.7)	40.0 (39.0–40.7)	39.6 (39.0–40.4)
Weight (kg)	292	3.5 (3.2–3.9)	3.5 (3.3–3.9)	3.5 (3.2–3.9)
Length (cm)	284	51.0 (50.0–53.0)	52.0 (50.5–53.0)	51.0 (50.0–53.0)
Triceps + subscapular skinfold thickness sum (mm)	282	9.6 (8.2–11.1)	9.2 (8.1–10.9)	9.7 (8.2–11.2)
Head circumference (cm)	283	35.0 (34.0–35.5)	34.5 (34.0–35.5)	35.0 (34.0–35.8)
**6-wk time point**				
(Median 6.0 wk; range 3.3, 11.1)				
Age at visit (wk)	290	6.0 (5.3–7.1)	6.0 (5.3–6.8)	6.0 (5.3–7.1)
Weight (kg)	289	4.8 (4.3–5.4)	4.8 (4.3–5.4)	4.7 (4.4–5.4)
Length (cm)	285	55.5 (54.0–57.0)	55.5 (54.0–57.0)	55.5 (54.0–57.0)
Triceps + subscapular skinfold thickness sum (mm)	289	12.0 (10.6–13.8)	11.6 (10.5–13.7)	12.1 (10.7–13.9)
Head circumference (cm)	291	38.0 (37.0–39.0)	37.9 (37.0–38.9)	38.0 (37.0–39.0)
**6-mo time point**				
(Median 6.6 mo; range 5.9, 8.2)				
Age at visit (mo)	271	6.6 (6.4–6.9)	6.7 (6.4–6.9)	6.6 (6.4–6.9)
Weight (kg)	271	7.9 (7.3–8.6)	8.1 (7.5-8.6)	7.9 (7.2–8.6)
Length (cm)	267	68.5 (66.5–70.0)	69.0 (67.4–70.1)	68.0 (66.0–70.0)
Triceps + subscapular skinfold thickness sum (mm)	267	15.8 (13.9–18.0)	16.0 (14.1–18.4)	15.7 (13.8–18.0)
Head circumference (cm)	270	43.7 (42.8–44.6)	43.7 (42.8–44.3)	43.6 (42.8–44.6)
**12-mo time point**				
(Median 12.8 mo; range 11.7, 16.5)				
Age at visit (mo)	267	12.8 (12.5–13.3)	12.7 (12.5–13.0)	12.8 (12.4–13.4)
Weight (kg)	267	10.0 (9.2–10.8)	10.1 (9.3–10.7)	9.9 (9.2–10.8)
Length (cm)	259	75.5 (73.5–77.5)	74.9 (73.4–77.5)	75.5 (73.5–77.5)
Triceps + subscapular skinfold thickness sum (mm)	263	16.0 (14.2–18.7)	16.9 (14.8–19.0)	15.9 (14.0–18.7)
Head circumference (cm)	267	46.5 (45.2–47.3)	45.9 (45.0–46.8)	46.5 (45.5–47.5)
**4-y time point**				
(Median 4.1 y; range 3.9, 5.5)				
Age at visit (y)	233	4.1 (4.0–4.3)	4.1 (4.1–4.2)	4.1 (4.0–4.3)
Weight (kg)	205	17.6 (16.3–19.5)	17.9 (16.8–20.0)	17.6 (16.3–19.4)
Length (cm)wk	201	106.2 (103.4–109.1)	106.6 (103.8–109.2)	106.2 (103.2–109.0)
Triceps + subscapular skinfold thickness sum (mm)	205	14.9 (12.8–17.0)	15.4 (13.3–18.6)	14.7 (12.6–16.6)
Head circumference (cm)	233	51.0 (50.0–52.0)	51.0 (50.0–52.0)	51.0 (50.0–52.0)

Categorical variables reported as n (%); numerical variables reported as median (IQR).

### HMO concentrations and growth to 6 mo of age

**FIGURE 1:**
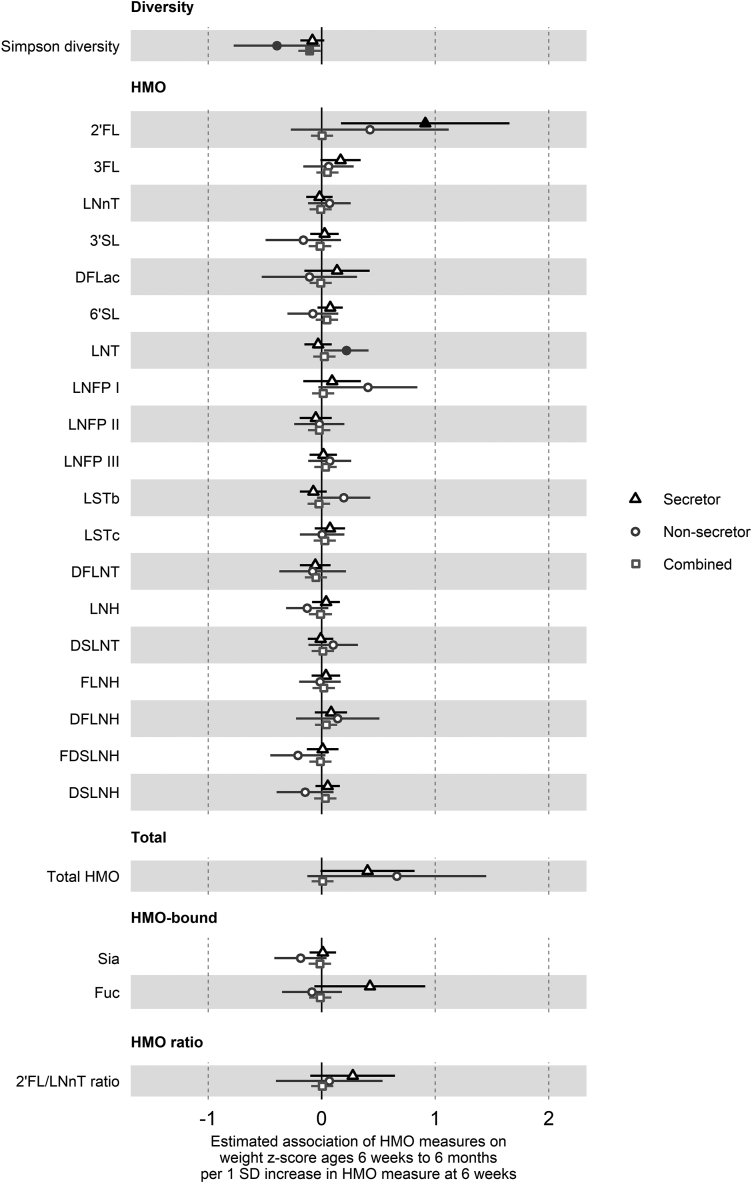
Estimated association of 1 SD increase in HMO measures on weight *z*-score at 6 wk and 6 mo of age. Forest plots of the estimated difference in weight *z*-score (SD units) at 6 wk and 6 mo of age/1 SD increase in HMO measure, from hierarchical mixed-effects linear models adjusted for weight *z*-score at birth and potential confounders. Secretor status-stratified models are depicted with triangles (secretor only, *n* = 221) and circles (nonsecretor only, *n* = 71), and combined models with the overall cohort are squares (*n* = 292). All HMO measures other than diversity were log-transformed prior to analysis. Error bars are 95% CI. Closed points represent *P* < 0.05. HMOs are the log concentrations of the 19 species of HMO measured. Diversity is the Shannon diversity of these HMOs. Total is the total concentration of the 19 HMOs. HMO-bound is the log concentrations of sia and fuc. 2’FL, 2’-fucosyllactose; 3’SL, 3’-sialyllactose; 3FL, 3-fucosyllactose; 6’SL, 6’-sialyllactose; DFLac, difucosyllactose; DFLNH, difucosyllacto-N-hexaose; DFLNT, difucosyllacto-N-tetraose; DSLNH, disialyllacto-N-hexaose; DSLNT, disialyllacto-N-tetraose; FDSLNH, fucodisialyllacto-N-hexaose; FLNH, fucosyllacto-N-hexaose; Fuc, HMO-bound fucose; HMO, human milk oligosaccharide; LNFP, lacto-N-fucopentaose; LNH, lacto-N-hexaose; LNnT, lacto-N-neotetraose; LNT, lacto-N-tetraose; LSTb, sialyl-lacto-N-tetraose b; LSTc, sialyl-lacto-N-tetraose c; Sia, HMO-bound sialic acid.

**FIGURE 2:**
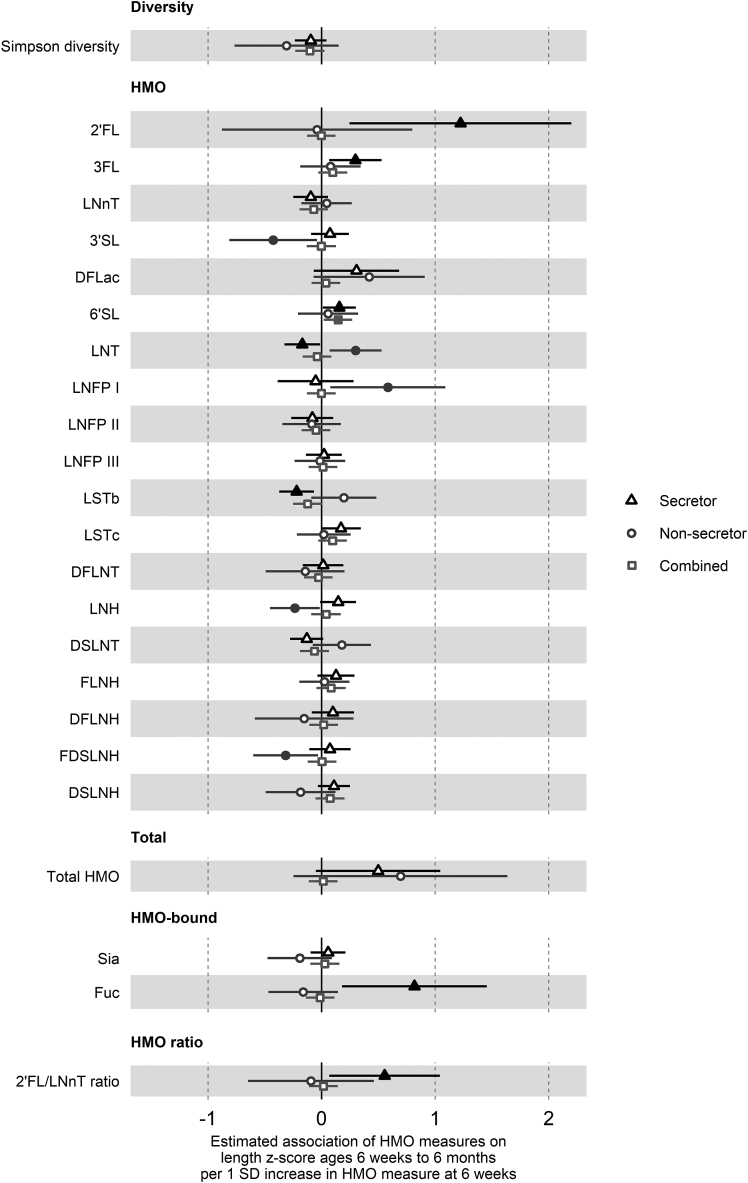
Estimated association of 1 SD increase in HMO measures on length *z*-score at 6 wk and 6 mo of age.Forest plots of the estimated difference in length *z*-score (SD units) at 6 wk and 6 mo of age/1 SD increase in HMO measure, from hierarchical mixed-effects linear models adjusted for length *z*-score at birth and potential confounders. Secretor status-stratified models are depicted with triangles (secretor only, *n* = 221) and circles (nonsecretor only, *n* = 69), and combined models with the overall cohort are squares (*n* = 290). All HMO measures other than diversity were log-transformed prior to analysis. Error bars are 95% CI. Closed points represent *P* < 0.05. HMOs are the log concentrations of the 19 species of HMO measured. Diversity is the Shannon diversity of these HMOs. Total is the total concentration of the 19 HMOs. HMO-bound is the log concentrations of sia and fuc. 2’FL, 2’-fucosyllactose; 3’SL, 3’-sialyllactose; 3FL, 3-fucosyllactose; 6’SL, 6’-sialyllactose; DFLac, difucosyllactose; DFLNH, difucosyllacto-N-hexaose; DFLNT, difucosyllacto-N-tetraose; DSLNH, disialyllacto-N-hexaose; DSLNT, disialyllacto-N-tetraose; FDSLNH, fucodisialyllacto-N-hexaose; FLNH, fucosyllacto-N-hexaose; Fuc, HMO-bound fucose; HMO, human milk oligosaccharide; LNFP, lacto-N-fucopentaose; LNH, lacto-N-hexaose; LNnT, lacto-N-neotetraose; LNT, lacto-N-tetraose; LSTb, sialyl-lacto-N-tetraose b; LSTc, sialyl-lacto-N-tetraose c; Sia, HMO-bound sialic acid.

As associations of HMO concentrations at 6 wk postpartum with child growth may be most evident with direct exposure during the lactation period, we first used hierarchical mixed-effects linear models clustered on the participant for change in anthropometric *z*-scores from birth at 6 wk and 6 mo (Supplementary Table 2 for overall cohort, Table 2 for secretor status-stratified models). Secretor status did not show evidence of association with any of the anthropometric *z*-scores (Supplementary Table 3). However, higher 2’FL concentrations were associated with greater weight and length *z*-scores at 6 wk and 6 mo in children born to secretor mothers only [weight: 0.91 SD higher weight *z*-score/1 SD log 2’FL, 95% CI (0.17, 1.65), *P* = 0.02, Figure 1; length: 1.22 SD (0.25, 2.20), *P* = 0.02, Figure 2]. In addition, the 2’FL/LNnT ratio was similarly associated with a greater length *z*-score in the secretor group only [0.55 SD (0.07, 1.04), *P* = 0.02].

**TABLE 2:** Summary of associations between human milk oligosaccharide measures and *z*-score outcomes at 6 wk to 6 mo of age, by secretor status

HMO measure	Length *z*-score (aged 6 wk to 6 mo)					
	Secretor group (*n* = 221)			Nonsecretor group (*n* = 69)		
	Estimate (β)	95% CI	*P* value	Estimate (β)	95% CI	*P* value
Simpson diversity	–0.096	–0.235, 0.043	0.177	–0.308	–0.768, 0.151	0.193
2’FL	1.223	0.246, 2.200	0.015	–0.040	–0.879, 0.799	0.925
3FL	0.297	0.066, 0.528	0.012	0.078	–0.187, 0.343	0.567
LNnT	–0.096	–0.249, 0.057	0.222	0.044	–0.178, 0.266	0.697
3’SL	0.073	–0.092, 0.239	0.385	–0.426	–0.812, –0.04	0.034
DFLac	0.307	–0.069, 0.683	0.112	0.420	–0.070, 0.909	0.098
6’SL	0.156	0.011, 0.301	0.036	0.057	–0.207, 0.322	0.673
LNT	–0.171	–0.328, –0.014	0.034	0.299	0.07, 0.528	0.013
LNFP I	–0.053	–0.387, 0.281	0.756	0.583	0.077, 1.089	0.027
LNFP II	–0.082	–0.267, 0.103	0.385	–0.087	–0.345, 0.170	0.509
LNFP III	0.021	–0.137, 0.179	0.799	–0.016	–0.238, 0.207	0.891
LSTb	–0.220	–0.373, –0.067	0.005	0.196	–0.091, 0.482	0.185
LSTc	0.170	–0.005, 0.346	0.059	0.019	–0.219, 0.256	0.879
DFLNT	0.014	–0.165, 0.192	0.882	–0.145	–0.492, 0.201	0.415
LNH	0.146	–0.013, 0.304	0.073	–0.236	–0.454, –0.017	0.039
DSLNT	–0.131	–0.278, 0.015	0.081	0.178	–0.078, 0.434	0.177
FLNH	0.127	–0.037, 0.290	0.130	0.024	–0.196, 0.245	0.829
DFLNH	0.099	–0.087, 0.285	0.299	–0.153	–0.588, 0.282	0.493
FDSLNH	0.074	–0.109, 0.256	0.431	–0.317	–0.602, –0.032	0.033
DSLNH	0.108	–0.033, 0.250	0.136	–0.185	–0.492, 0.121	0.241
Total HMO	0.497	–0.051, 1.046	0.077	0.695	–0.247, 1.637	0.153
HMO-bound Sia	0.057	–0.096, 0.210	0.466	–0.192	–0.475, 0.091	0.189
HMO-bound Fuc	0.817	0.179, 1.455	0.013	–0.162	–0.469, 0.144	0.304
2’FL/LNnT ratio	0.555	0.067, 1.042	0.027	–0.094	–0.648, 0.460	0.740

HMO measure	Weight *z*-score (aged 6 wk to 6 mo)					
	Secretor group (*n* = 221)			Nonsecretor group (*n* = 71)		
	Estimate (β)	95% CI	*P* value	Estimate (β)	95% CI	*P* value
Simpson diversity	–0.082	–0.188, 0.023	0.126	–0.395	–0.774, -0.015	0.046
2’FL	0.912	0.170, 1.654	0.017	0.424	–0.272, 1.120	0.237
3FL	0.165	–0.012, 0.342	0.069	0.060	–0.162, 0.282	0.600
LNnT	–0.020	–0.137, 0.097	0.743	0.068	–0.120, 0.255	0.482
3’SL	0.025	–0.101, 0.151	0.701	–0.162	–0.494, 0.171	0.345
DFLac	0.135	–0.153, 0.422	0.360	–0.109	–0.529, 0.312	0.614
6’SL	0.074	–0.037, 0.186	0.192	–0.078	–0.301, 0.145	0.497
LNT	–0.032	–0.153, 0.089	0.605	0.217	0.021, 0.414	0.034
LNFP I	0.091	–0.162, 0.344	0.482	0.406	–0.029, 0.842	0.072
LNFP II	–0.053	–0.193, 0.088	0.462	–0.020	–0.241, 0.200	0.856
LNFP III	0.015	–0.106, 0.135	0.814	0.070	–0.118, 0.259	0.466
LSTb	–0.074	–0.192, 0.044	0.220	0.194	–0.041, 0.429	0.111
LSTc	0.073	–0.061, 0.207	0.290	0.004	–0.193, 0.200	0.970
DFLNT	–0.057	–0.193, 0.079	0.410	–0.080	–0.374, 0.214	0.595
LNH	0.038	–0.085, 0.160	0.545	–0.128	–0.314, 0.059	0.184
DSLNT	–0.010	–0.121, 0.102	0.864	0.101	–0.117, 0.319	0.368
FLNH	0.037	–0.088, 0.161	0.565	–0.014	–0.198, 0.169	0.879
DFLNH	0.082	–0.060, 0.223	0.259	0.141	–0.226, 0.508	0.454
FDSLNH	0.009	–0.130, 0.147	0.903	–0.210	–0.453, 0.033	0.096
DSLNH	0.053	–0.055, 0.161	0.338	–0.147	–0.398, 0.104	0.255
Total HMO	0.403	–0.011, 0.818	0.058	0.662	–0.126, 1.450	0.105
HMO-bound Sia	0.010	–0.107, 0.126	0.872	–0.187	–0.417, 0.044	0.117
HMO-bound Fuc	0.424	–0.064, 0.913	0.090	–0.086	–0.349, 0.177	0.525
2’FL/LNnT ratio	0.273	–0.101, 0.646	0.154	0.067	–0.403, 0.536	0.782

HMO measure	Weight-for-length *z*-score (aged 6 wk to 6 mo)					
	Secretor group (*n* = 221)			Nonsecretor group (*n* = 69)		
	Estimate (β)	95% CI	*P* value	Estimate (β)	95% CI	*P* value
Simpson diversity	–0.028	–0.175, 0.119	0.708	–0.169	–0.699, 0.362	0.536
2’FL	–0.056	–1.101, 0.988	0.916	0.482	–0.461, 1.426	0.320
3FL	–0.137	–0.384, 0.110	0.278	–0.032	–0.336, 0.272	0.839
LNnT	0.073	–0.089, 0.235	0.378	0.046	–0.207, 0.300	0.722
3’SL	–0.092	–0.266, 0.082	0.303	0.344	–0.100, 0.789	0.134
DFLac	–0.301	–0.697, 0.095	0.137	–0.635	–1.182, -0.088	0.026
6’SL	–0.103	–0.257, 0.052	0.194	–0.185	–0.484, 0.114	0.231
LNT	0.151	–0.015, 0.317	0.077	–0.062	–0.338, 0.214	0.659
LNFP I	0.237	–0.114, 0.588	0.187	–0.172	–0.777, 0.432	0.578
LNFP II	0.001	–0.194, 0.196	0.990	0.088	–0.209, 0.385	0.562
LNFP III	0.040	–0.125, 0.206	0.634	0.135	–0.121, 0.390	0.307
LSTb	0.135	–0.028, 0.298	0.106	0.064	–0.270, 0.397	0.709
LSTc	–0.087	–0.273, 0.099	0.360	–0.035	–0.304, 0.235	0.802
DFLNT	–0.123	–0.311, 0.065	0.201	0.055	–0.34, 0.451	0.786
LNH	–0.032	–0.199, 0.135	0.708	0.073	–0.186, 0.332	0.584
DSLNT	0.148	–0.005, 0.302	0.060	–0.072	–0.369, 0.226	0.638
FLNH	–0.041	–0.212, 0.130	0.640	–0.063	–0.316, 0.189	0.624
DFLNH	0.059	–0.137, 0.256	0.556	0.437	–0.046, 0.919	0.081
FDSLNH	–0.054	–0.246, 0.138	0.582	0.091	–0.25, 0.431	0.604
DSLNH	–0.054	–0.204, 0.096	0.481	0.030	–0.323, 0.383	0.868
Total HMO	0.047	–0.535, 0.629	0.875	0.024	–1.072, 1.120	0.966
HMO-bound Sia	–0.060	–0.222, 0.102	0.471	–0.034	–0.359, 0.292	0.839
HMO-bound Fuc	–0.387	–1.067, 0.294	0.267	0.086	–0.269, 0.44	0.638
2’FL/LNnT ratio	–0.215	–0.737, 0.306	0.419	0.129	–0.497, 0.756	0.687

HMO measure	Head circumference *z*-score (aged 6 wk to 6 mo)					
	Secretor group (*n* = 221)			Nonsecretor group (*n* = 71)		
	Estimate (β)	95% CI	*P* value	Estimate (β)	95% CI	*P* value
Simpson diversity	–0.066	–0.217, 0.086	0.397	–0.138	–0.452, 0.177	0.394
2’FL	0.637	–0.442, 1.716	0.249	0.685	0.140, 1.229	0.016
3FL	–0.034	–0.294, 0.226	0.795	0.092	–0.085, 0.269	0.312
LNnT	0.107	–0.063, 0.276	0.218	–0.031	–0.182, 0.120	0.686
3’SL	0.080	–0.102, 0.261	0.392	0.096	–0.174, 0.365	0.489
DFLac	–0.052	–0.472, 0.368	0.808	–0.105	–0.444, 0.233	0.543
6’SL	–0.170	–0.329, –0.010	0.038	–0.205	–0.379, –0.032	0.024
LNT	0.015	–0.159, 0.188	0.869	0.049	–0.113, 0.21	0.556
LNFP I	0.174	–0.193, 0.541	0.352	0.348	–0.002, 0.697	0.055
LNFP II	0.023	–0.180, 0.227	0.823	0.118	–0.057, 0.293	0.190
LNFP III	0.039	–0.135, 0.213	0.661	0.101	–0.050, 0.252	0.194
LSTb	0.004	–0.164, 0.172	0.965	0.055	–0.139, 0.248	0.582
LSTc	0.022	–0.174, 0.218	0.827	–0.123	–0.278, 0.032	0.125
DFLNT	–0.054	–0.252, 0.144	0.596	0.149	–0.083, 0.382	0.213
LNH	0.040	–0.133, 0.213	0.652	–0.019	–0.171, 0.134	0.812
DSLNT	–0.043	–0.204, 0.119	0.606	–0.129	–0.302, 0.044	0.149
FLNH	–0.025	–0.204, 0.153	0.782	0.003	–0.146, 0.151	0.973
DFLNH	0.001	–0.204, 0.206	0.989	–0.068	–0.364, 0.228	0.654
FDSLNH	–0.052	–0.253, 0.150	0.616	–0.003	–0.202, 0.197	0.980
DSLNH	–0.134	–0.291, 0.023	0.096	–0.233	–0.429, –0.037	0.023
Total HMO	0.501	–0.102, 1.103	0.105	0.173	–0.472, 0.818	0.601
HMO-bound Sia	–0.121	–0.289, 0.048	0.161	–0.229	–0.410, –0.049	0.016
HMO-bound Fuc	0.257	–0.457, 0.97	0.482	0.145	–0.063, 0.354	0.177
2’FL/LNnT ratio	–0.140	–0.682, 0.402	0.614	0.345	–0.022, 0.712	0.070

HMO measure	Sum of skinfold thickness *z*-score (aged 6 wk to 6 mo)					
	Secretor group (*n* = 221)			Nonsecretor group (*n* = 71)		
	Estimate (β)	95% CI	*P* value	Estimate (β)	95% CI	*P* value
Simpson diversity	–0.025	–0.164, 0.114	0.724	–0.516	–0.985, –0.048	0.035
2’FL	0.016	–0.975, 1.007	0.975	0.399	–0.461, 1.258	0.367
3FL	0.217	–0.017, 0.452	0.071	0.104	–0.167, 0.375	0.454
LNnT	0.052	–0.103, 0.206	0.514	–0.023	–0.253, 0.208	0.846
3’SL	0.107	–0.058, 0.273	0.205	0.179	–0.228, 0.585	0.392
DFLac	0.103	–0.280, 0.485	0.600	–0.141	–0.657, 0.374	0.593
6’SL	–0.160	–0.305, –0.014	0.033	–0.302	–0.568, –0.037	0.029
LNT	–0.017	–0.176, 0.143	0.838	–0.072	–0.319, 0.175	0.570
LNFP I	–0.148	–0.484, 0.187	0.386	–0.107	–0.651, 0.437	0.702
LNFP II	0.083	–0.103, 0.268	0.385	0.076	–0.194, 0.346	0.585
LNFP III	0.040	–0.118, 0.199	0.621	0.152	–0.078, 0.383	0.200
LSTb	0.067	–0.087, 0.221	0.393	0.059	–0.236, 0.354	0.695
LSTc	–0.121	–0.299, 0.058	0.186	–0.113	–0.352, 0.125	0.356
DFLNT	–0.002	–0.183, 0.178	0.980	0.096	–0.262, 0.454	0.600
LNH	0.048	–0.111, 0.207	0.555	–0.025	–0.257, 0.208	0.835
DSLNT	0.059	–0.088, 0.207	0.432	–0.001	–0.269, 0.267	0.993
FLNH	–0.105	–0.268, 0.057	0.207	–0.081	–0.305, 0.143	0.483
DFLNH	–0.121	–0.307, 0.066	0.206	0.339	–0.107, 0.784	0.141
FDSLNH	0.067	–0.117, 0.251	0.475	0.041	–0.265, 0.347	0.792
DSLNH	–0.073	–0.217, 0.07	0.318	–0.132	–0.440, 0.177	0.406
Total HMO	–0.087	–0.641, 0.467	0.758	–0.219	–1.202, 0.763	0.663
HMO-bound Sia	0.014	–0.14, 0.168	0.859	–0.103	–0.391, 0.184	0.483
HMO-bound Fuc	–0.265	–0.915, 0.386	0.426	0.080	–0.243, 0.403	0.631
2’FL/LNnT ratio	–0.130	–0.624, 0.364	0.607	0.202	–0.369, 0.773	0.490

Estimates are average change in outcome *z*-score/1 SD change in Simpson diversity, or 1 SD change in log concentrations of individual HMOs, total HMO, fuc, and sia, or 1 SD change in the log-ratio of 2'FL and LNnT concentrations. Models were hierarchical mixed-effects linear models adjusted for maternal prepregnancy BMI, household income during pregnancy, infant sex, human milk feeding duration in completed weeks (up to 6 mo), any introduction to formula milk feeding by 6 mo, and the corresponding *z*-score at birth. All models include an interaction term between HMO and time and use an unstructured correlation structure and a random intercept for each participant.

2’FL, 2’-fucosyllactose; 3’SL, 3’-sialyllactose; 3FL, 3-fucosyllactose; 6’SL, 6’-sialyllactose; DFLac, difucosyllactose; DFLNH, difucosyllacto-N-hexaose; DFLNT, difucosyllacto-N-tetraose; DSLNH, disialyllacto-N-hexaose; DSLNT, disialyllacto-N-tetraose; FDSLNH, fucodisialyllacto-N-hexaose; FLNH, fucosyllacto-N-hexaose; Fuc, HMO-bound fucose; HMO, human milk oligosaccharide; LNFP, lacto-N-fucopentaose; LNH, lacto-N-hexaose; LNnT, lacto-N-neotetraose; LNT, lacto-N-tetraose; LSTb, sialyl-lacto-N-tetraose b; LSTc, sialyl-lacto-N-tetraose c; Sia, HMO-bound sialic acid.

**FIGURE 3:**
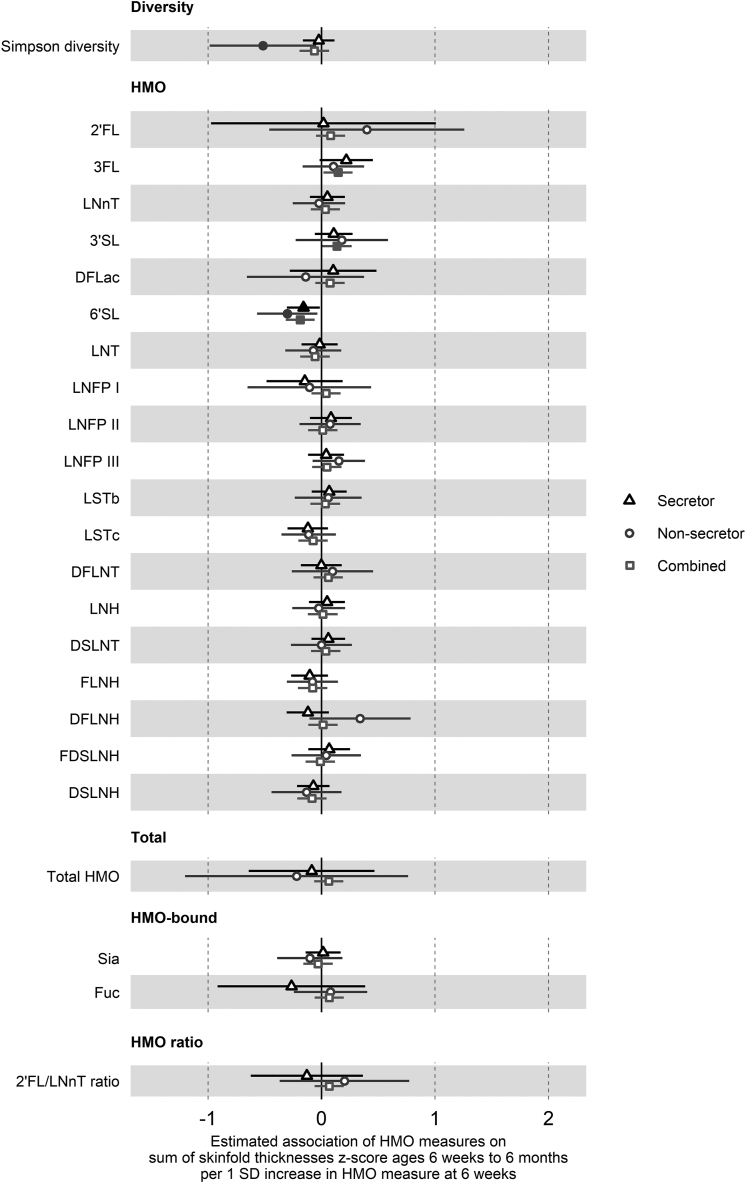
Estimated association of 1 SD increase in HMO measures on the sum of skinfold thickness *z*-score at 6 wk and 6 mo of age.Forest plots of the estimated difference in the sum of skinfold thickness *z*-score (SD units) at 6 wk and 6 mo of age/1 SD increase in HMO measure, from hierarchical mixed-effects linear models adjusted for the sum of skinfold thickness *z*-score at birth and potential confounders. Secretor status-stratified models are depicted with triangles (secretor only, *n* = 221) and circles (nonsecretor only, *n* = 71), and combined models with the overall cohort are squares (*n* = 292). All HMO measures other than diversity were log-transformed prior to analysis. Error bars are 95% CI. Closed points represent *P* < 0.05. HMOs are the log concentrations of the 19 species of HMO measured. Diversity is the Shannon diversity of these HMOs. Total is the total concentration of the 19 HMOs. HMO-bound is the log concentrations of sia and fuc. 2’FL, 2’-fucosyllactose; 3’SL, 3’-sialyllactose; 3FL, 3-fucosyllactose; 6’SL, 6’-sialyllactose; DFLac, difucosyllactose; DFLNH, difucosyllacto-N-hexaose; DFLNT, difucosyllacto-N-tetraose; DSLNH, disialyllacto-N-hexaose; DSLNT, disialyllacto-N-tetraose; FDSLNH, fucodisialyllacto-N-hexaose; FLNH, fucosyllacto-N-hexaose; Fuc, HMO-bound fucose; HMO, human milk oligosaccharide; LNFP, lacto-N-fucopentaose; LNH, lacto-N-hexaose; LNnT, lacto-N-neotetraose; LNT, lacto-N-tetraose; LSTb, sialyl-lacto-N-tetraose b; LSTc, sialyl-lacto-N-tetraose c; Sia, HMO-bound sialic acid.

**FIGURE 4:**
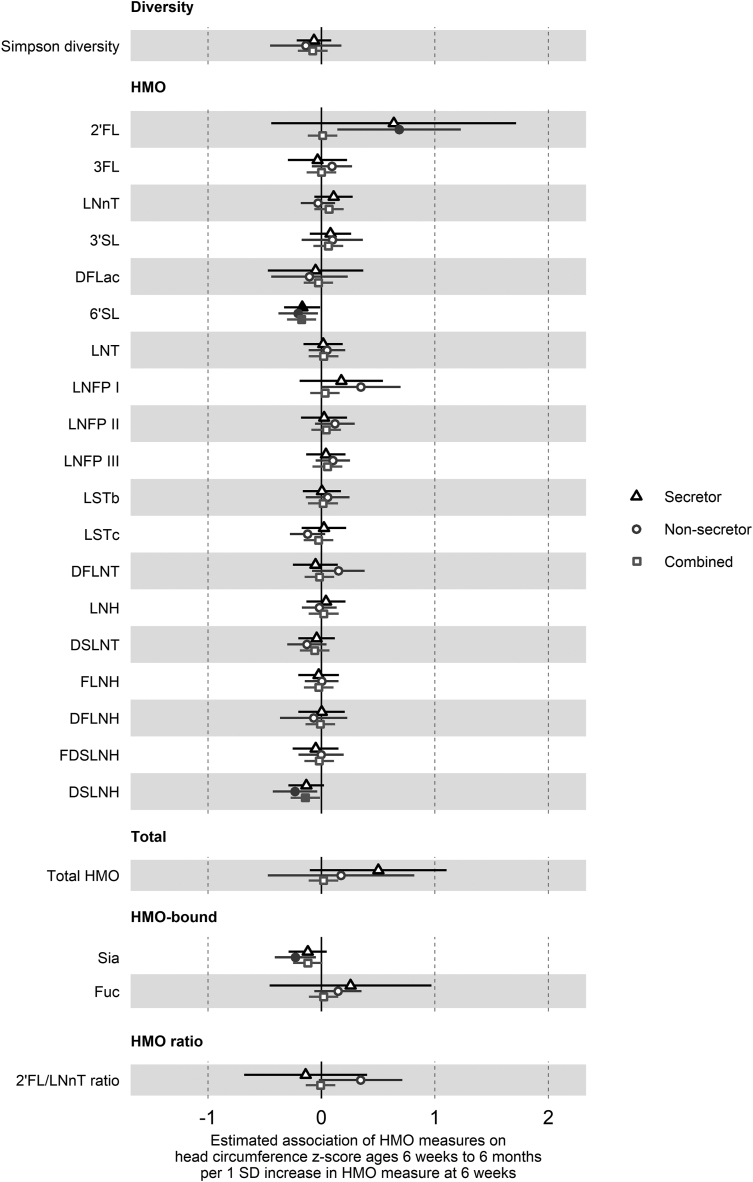
Estimated association of 1 SD increase in HMO measures on head circumference *z*-score at 6 wk and 6 mo of age. Forest plots of the estimated difference in head circumference *z*-score (SD units) at 6 wk and 6 mo of age/1 SD increase in HMO measure, from hierarchical mixed-effects linear models adjusted for head circumference *z*-score at birth and potential confounders. Secretor status-stratified models are depicted with triangles (secretor only, *n* = 221) and circles (nonsecretor only, *n* = 71), and combined models with the overall cohort are squares (*n* = 292). All HMO measures other than diversity were log-transformed prior to analysis. Error bars are 95% CI. Closed points represent *P* < 0.05. HMOs are the log concentrations of the 19 species of HMO measured. Diversity is the Shannon diversity of these HMOs. Total is the total concentration of the 19 HMOs. HMO-bound is the log concentrations of sia and fuc. 2’FL, 2’-fucosyllactose; 3’SL, 3’-sialyllactose; 3FL, 3-fucosyllactose; 6’SL, 6’-sialyllactose; DFLac, difucosyllactose; DFLNH, difucosyllacto-N-hexaose; DFLNT, difucosyllacto-N-tetraose; DSLNH, disialyllacto-N-hexaose; DSLNT, disialyllacto-N-tetraose; FDSLNH, fucodisialyllacto-N-hexaose; FLNH, fucosyllacto-N-hexaose; Fuc, HMO-bound fucose; HMO, human milk oligosaccharide; LNFP, lacto-N-fucopentaose; LNH, lacto-N-hexaose; LNnT, lacto-N-neotetraose; LNT, lacto-N-tetraose; LSTb, sialyl-lacto-N-tetraose b; LSTc, sialyl-lacto-N-tetraose c Sia, HMO-bound sialic acid.

In the overall cohort (not stratified by secretor status), lower HMO diversity was associated with greater weight *z*-score [–0.11 SD (–0.21, –0.01), *P* = 0.03] (Figure 1), higher 3FL and 3’SL and lower 6’SL with greater skinfold thickness *z*-score [3FL: 0.15 SD (0.02, 0.27), *P* = 0.03; 3’SL: 0.14 SD (0.01, 0.27), *P* = 0.04; 6’SL: −0.19 SD (−0.32, −0.06), *P* = 0.004] (Figure 3), and lower 6’SL and DSLNH were associated with greater head circumference *z*-score [6’SL: −0.18 SD (−0.30, −0.05), *P* = 0.008; DSLNH: −0.14 SD (−0.27, −0.01), *P* = 0.03] (Figure 4).

**FIGURE 5:**
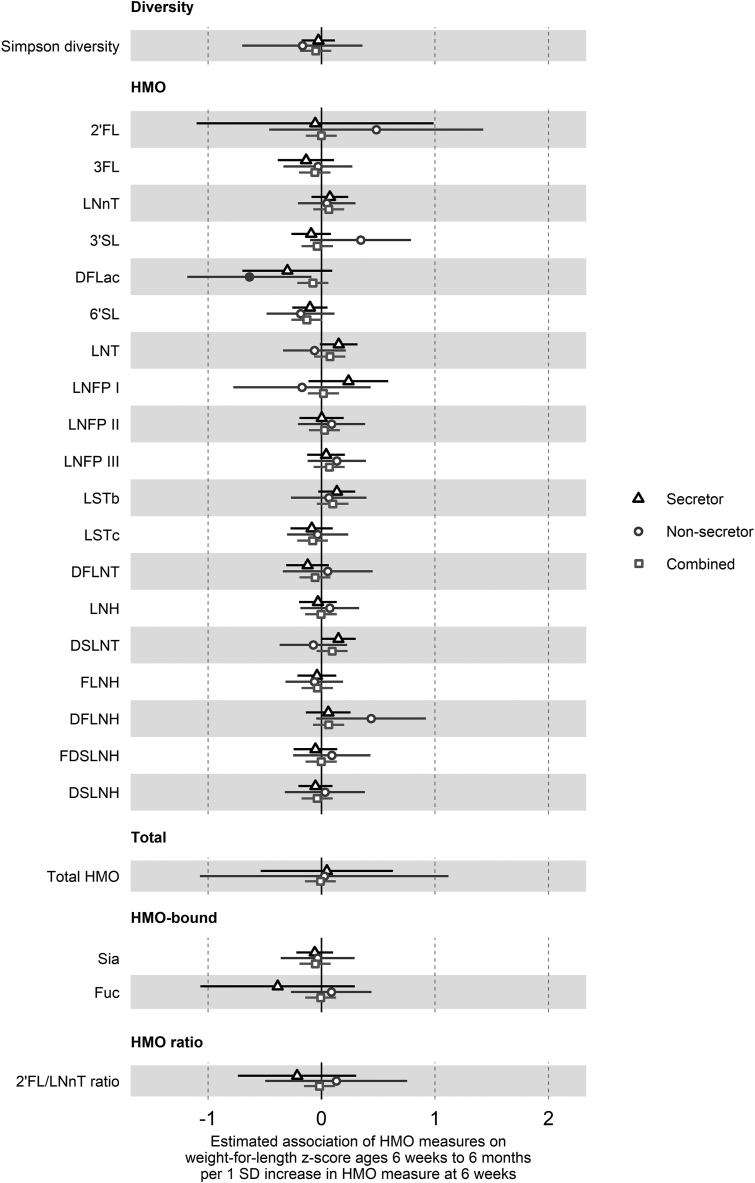
Estimated association of 1 SD increase in HMO measures on weight-for-length *z*-score at 6 wk and 6 mo of age. Forest plots of the estimated difference in weight-for-length *z*-score (SD units) at 6 wk and 6 mo of age/1 SD increase in HMO measure, from hierarchical mixed-effects linear models adjusted for weight-for-length *z*-score at birth and potential confounders. Secretor status-stratified models are depicted with triangles (secretor only, *n* = 221) and circles (nonsecretor only, *n* = 69), and combined models with the overall cohort are squares (*n* = 290). All HMO measures other than diversity were log-transformed prior to analysis. Error bars are 95% CI. Closed points represent *P* < 0.05. HMOs are the log concentrations of the 19 species of HMO measured. Diversity is the Shannon diversity of these HMOs. Total is the total concentration of the 19 HMOs. HMO-bound is the log concentrations of sia and fuc. 2’FL, 2’-fucosyllactose; 3’SL, 3’-sialyllactose; 3FL, 3-fucosyllactose; 6’SL, 6’-sialyllactose; DFLac, difucosyllactose; DFLNH, difucosyllacto-N-hexaose; DFLNT, difucosyllacto-N-tetraose; DSLNH, disialyllacto-N-hexaose; DSLNT, disialyllacto-N-tetraose; FDSLNH, fucodisialyllacto-N-hexaose; FLNH, fucosyllacto-N-hexaose; Fuc, HMO-bound fucose; HMO, human milk oligosaccharide; LNFP, lacto-N-fucopentaose; LNH, lacto-N-hexaose; LNnT, lacto-N-neotetraose; LNT, lacto-N-tetraose; LSTb, sialyl-lacto-N-tetraose b; LSTc, sialyl-lacto-N-tetraose c; Sia, HMO-bound sialic acid.

Other associations observed only in infants born to secretor mothers were higher 3FL, 6’SL, and fuc and lower LNT and LSTb levels with greater length *z*-score (Figure 2). In children born to nonsecretor mothers, higher LNT was associated with greater weight *z*-score (Figure 1), higher LNT and LNFP I and lower 3’SL, LNH, and FDSLNH with greater length *z*-score (Figure 2), lower HMO diversity with greater skinfold thickness *z*-score (Figure 3), higher 2’FL and lower sia with greater head circumference *z*-score (Figure 4), and lower difucosyllactose with greater weight-for-length z-score (Figure 5). Additional adjustments for introduction to solid food or the week of introduction to formula feeding did not change findings (data not shown).

There was no clear evidence for differences by sex in the associations between HMO concentration and any of the outcomes at 6 wk and 6 mo (data not shown).

### HMO concentrations and growth from 12 mo to 4 y of age

To investigate whether HMO concentration at 6 wk postpartum has a persistent association with offspring growth after the introduction of complementary foods, hierarchical mixed-effects linear models with anthropometric *z*-scores at 12 mo and 4 y of age as the outcome were investigated (Supplementary Table 4 for overall cohort, Table 3 for secretor status-stratified models). In contrast to the models to 6 mo of age, 2’FL was not strongly associated with weight or length in the secretor group (Supplementary Figures 4 and 5). However, higher 2’FL was associated with greater head circumference *z*-score [0.15 SD (0.02, 0.29), *P* = 0.03] (Supplementary Figure 6) in the overall cohort, and higher weight-for-length *z*-score [1.16 SD (0.02, 2.30), *P* = 0.05] (Supplementary Figure 7) for children in the secretor group.

**TABLE 3:** Summary of associations between human milk oligosaccharide measures and *z*-score outcomes at 12 mo to 4 y of age, by secretor status

HMO measure	Length *z*-score (aged 12 mo to 4 y)					
	Secretor group (*n* = 199)			Nonsecretor group (*n* = 66)		
	Estimate (β)	95% CI	*P* value	Estimate (β)	95% CI	*P* value
Simpson diversity	0.087	–0.085, 0.260	0.321	0.100	–0.526, 0.726	0.755
2’FL	–0.326	–1.542, 0.891	0.600	–0.224	–1.311, 0.862	0.687
3FL	0.262	–0.043, 0.567	0.094	–0.190	–0.531, 0.150	0.278
LNnT	–0.119	–0.314, 0.076	0.232	0.157	–0.138, 0.452	0.301
3’SL	0.060	–0.144, 0.264	0.568	–0.378	–0.917, 0.161	0.174
DFLac	0.835	0.394, 1.276	0.000	–0.057	–0.716, 0.601	0.865
6’SL	0.157	–0.021, 0.336	0.086	–0.086	–0.430, 0.258	0.625
LNT	–0.079	–0.269, 0.110	0.414	–0.073	–0.375, 0.229	0.638
LNFP I	–0.349	–0.764, 0.066	0.101	–0.188	–0.852, 0.477	0.582
LNFP II	0.131	–0.097, 0.36	0.262	0.211	–0.117, 0.539	0.213
LNFP III	0.080	–0.121, 0.281	0.436	0.202	–0.085, 0.49	0.173
LSTb	–0.094	–0.280, 0.091	0.320	–0.165	–0.531, 0.200	0.378
LSTc	0.136	–0.083, 0.355	0.224	–0.002	–0.302, 0.297	0.987
DFLNT	0.161	–0.064, 0.385	0.162	0.083	–0.366, 0.532	0.718
LNH	0.154	–0.041, 0.349	0.122	0.062	–0.227, 0.351	0.677
DSLNT	–0.050	–0.231, 0.132	0.591	0.095	–0.241, 0.431	0.583
FLNH	0.048	–0.151, 0.246	0.636	–0.394	–0.670, –0.119	0.007
DFLNH	–0.103	–0.338, 0.132	0.390	–0.619	–1.190, –0.048	0.038
FDSLNH	0.166	–0.058, 0.391	0.148	0.088	–0.290, 0.466	0.649
DSLNH	0.174	0.002, 0.345	0.049	0.059	–0.321, 0.439	0.761
Total HMO	–0.195	–0.869, 0.479	0.571	–0.387	–1.600, 0.826	0.534
HMO-bound Sia	0.160	–0.031, 0.351	0.103	0.038	–0.328, 0.404	0.841
HMO-bound Fuc	0.213	–0.568, 0.995	0.593	0.115	–0.282, 0.512	0.573
2’FL/LNnT ratio	0.225	–0.380, 0.830	0.467	–0.344	–1.075, 0.386	0.359

HMO measure	Weight *z*-score (aged 12 mo to 4 y)					
	Secretor group (*n* = 201)			Nonsecretor group (*n* = 66)		
	Estimate (β)	95% CI	*P* value	Estimate (β)	95% CI	*P* value
Simpson diversity	–0.086	–0.235, 0.063	0.258	–0.017	–0.544, 0.51	0.949
2’FL	0.816	–0.228, 1.861	0.127	0.587	–0.319, 1.493	0.209
3FL	0.108	–0.156, 0.372	0.422	–0.082	–0.369, 0.205	0.578
LNnT	–0.066	–0.234, 0.101	0.437	0.120	–0.126, 0.366	0.343
3’SL	0.089	–0.087, 0.265	0.324	0.272	–0.189, 0.733	0.252
DFLac	0.183	–0.213, 0.578	0.366	–0.011	–0.564, 0.543	0.970
6’SL	–0.034	–0.190, 0.123	0.675	0.006	–0.283, 0.295	0.968
LNT	–0.102	–0.269, 0.064	0.230	–0.017	–0.272, 0.239	0.899
LNFP I	–0.126	–0.491, 0.239	0.499	0.334	–0.222, 0.890	0.244
LNFP II	0.060	–0.142, 0.261	0.562	–0.104	–0.382, 0.174	0.466
LNFP III	0.046	–0.126, 0.219	0.599	–0.141	–0.384, 0.101	0.258
LSTb	–0.059	–0.222, 0.103	0.477	0.124	–0.188, 0.436	0.440
LSTc	–0.074	–0.266, 0.118	0.450	–0.191	–0.439, 0.056	0.136
DFLNT	–0.063	–0.262, 0.135	0.532	–0.122	–0.499, 0.255	0.528
LNH	0.118	–0.053, 0.289	0.178	–0.054	–0.301, 0.193	0.670
DSLNT	–0.045	–0.204, 0.113	0.574	0.147	–0.134, 0.427	0.309
FLNH	–0.054	–0.226, 0.119	0.543	–0.048	–0.293, 0.197	0.703
DFLNH	–0.009	–0.213, 0.194	0.928	–0.563	–1.047, –0.079	0.026
FDSLNH	0.103	–0.094, 0.300	0.308	–0.073	–0.393, 0.247	0.656
DSLNH	–0.003	–0.155, 0.150	0.974	–0.041	–0.361, 0.278	0.800
Total HMO	0.319	–0.275, 0.912	0.294	–0.134	–1.153, 0.886	0.798
HMO-bound Sia	0.020	–0.147, 0.187	0.817	0.184	–0.120, 0.488	0.239
HMO-bound Fuc	0.451	–0.234, 1.136	0.199	–0.127	–0.46, 0.206	0.457
2’FL/LNnT ratio	0.378	–0.142, 0.898	0.156	0.072	–0.544, 0.687	0.820

HMO measure	Weight-for-length *z*-score (aged 12 mo to 4 y)					
	Secretor group (*n* = 199)			Nonsecretor group (*n* = 66)		
	Estimate (β)	95% CI	*P* value	Estimate (β)	95% CI	*P* value
Simpson diversity	–0.152	–0.314, 0.009	0.066	0.004	–0.634, 0.642	0.990
2’FL	1.157	0.018, 2.295	0.048	0.945	–0.157, 2.046	0.098
3FL	–0.057	–0.349, 0.236	0.705	0.037	–0.314, 0.388	0.837
LNnT	0.029	–0.158, 0.216	0.761	0.038	–0.262, 0.339	0.804
3’SL	0.068	–0.126, 0.262	0.491	0.598	0.052, 1.144	0.036
DFLac	–0.210	–0.645, 0.224	0.344	0.081	–0.59, 0.753	0.813
6’SL	–0.136	–0.307, 0.035	0.121	0.084	–0.267, 0.436	0.639
LNT	–0.071	–0.251, 0.108	0.437	0.018	–0.291, 0.328	0.908
LNFP I	0.004	–0.393, 0.401	0.984	0.573	–0.095, 1.240	0.098
LNFP II	0.032	–0.186, 0.249	0.775	–0.258	–0.591, 0.075	0.134
LNFP III	0.026	–0.166, 0.217	0.794	–0.303	–0.591, -0.015	0.044
LSTb	–0.061	–0.238, 0.116	0.500	0.218	–0.156, 0.592	0.259
LSTc	–0.202	–0.409, 0.005	0.057	–0.220	–0.518, 0.078	0.154
DFLNT	–0.193	–0.405, 0.019	0.076	–0.206	–0.661, 0.249	0.379
LNH	0.040	–0.146, 0.225	0.676	–0.056	–0.354, 0.242	0.715
DSLNT	–0.023	–0.196, 0.149	0.793	0.135	–0.205, 0.475	0.439
FLNH	–0.105	–0.294, 0.084	0.276	0.177	–0.118, 0.471	0.244
DFLNH	0.037	–0.185, 0.259	0.745	–0.399	–1.002, 0.204	0.200
FDSLNH	0.053	–0.162, 0.268	0.631	–0.114	–0.501, 0.273	0.564
DSLNH	–0.117	–0.281, 0.048	0.167	–0.060	–0.447, 0.326	0.760
Total HMO	0.518	–0.116, 1.152	0.111	0.040	–1.194, 1.273	0.950
HMO-bound Sia	–0.068	–0.251, 0.115	0.468	0.254	–0.114, 0.622	0.181
HMO-bound Fuc	0.417	–0.324, 1.157	0.271	–0.222	–0.625, 0.180	0.283
2’FL/LNnT ratio	0.223	–0.352, 0.799	0.448	0.367	–0.381, 1.115	0.340

HMO measure	Head circumference *z*-score (aged 12 mo to 4 y)					
	Secretor group (*n* = 210)			Nonsecretor group (*n* = 69)		
	Estimate (β)	95% CI	*P* value	Estimate (β)	95% CI	*P* value
Simpson diversity	–0.043	–0.191, 0.105	0.571	0.731	0.236, 1.225	0.005
2’FL	0.299	–0.748, 1.345	0.576	1.049	0.169, 1.930	0.023
3FL	0.017	–0.245, 0.280	0.898	0.096	–0.190, 0.383	0.513
LNnT	–0.034	–0.200, 0.133	0.693	–0.115	–0.358, 0.128	0.358
3’SL	0.031	–0.145, 0.207	0.731	0.280	–0.168, 0.728	0.225
DFLac	–0.108	–0.503, 0.288	0.594	–0.316	–0.864, 0.232	0.263
6’SL	–0.086	–0.242, 0.069	0.278	0.150	–0.138, 0.438	0.312
LNT	0.023	–0.144, 0.189	0.789	–0.035	–0.290, 0.220	0.789
LNFP I	0.159	–0.203, 0.522	0.390	0.440	–0.111, 0.992	0.123
LNFP II	–0.017	–0.216, 0.183	0.868	0.062	–0.219, 0.343	0.666
LNFP III	–0.026	–0.198, 0.146	0.765	0.013	–0.233, 0.260	0.916
LSTb	–0.008	–0.170, 0.154	0.925	–0.083	–0.393, 0.226	0.600
LSTc	0.014	–0.177, 0.205	0.882	0.072	–0.181, 0.325	0.579
DFLNT	–0.118	–0.314, 0.079	0.242	0.075	–0.304, 0.454	0.700
LNH	–0.046	–0.217, 0.125	0.599	0.217	–0.024, 0.458	0.082
DSLNT	–0.052	–0.209, 0.105	0.520	–0.188	–0.465, 0.089	0.188
FLNH	–0.090	–0.262, 0.081	0.304	0.142	–0.100, 0.383	0.254
DFLNH	–0.036	–0.238, 0.166	0.727	–0.309	–0.791, 0.174	0.215
FDSLNH	–0.113	–0.309, 0.083	0.261	0.183	–0.134, 0.499	0.263
DSLNH	–0.073	–0.224, 0.078	0.344	0.217	–0.100, 0.534	0.185
Total HMO	0.314	–0.275, 0.903	0.297	–0.172	–1.19, 0.847	0.742
HMO-bound Sia	–0.081	–0.247, 0.085	0.339	0.202	–0.100, 0.504	0.194
HMO-bound Fuc	0.166	–0.519, 0.851	0.636	0.119	–0.216, 0.454	0.490
2’FL/LNnT ratio	0.162	–0.359, 0.683	0.543	0.647	0.060, 1.233	0.035

HMO measure	Sum of skinfold thickness *z*-score (aged 12 mo to 4 y)					
	Secretor group (*n* = 208)			Nonsecretor group (*n* = 69)		
	Estimate (β)	95% CI	*P* value	Estimate (β)	95% CI	*P* value
Simpson diversity	–0.042	–0.199, 0.116	0.604	0.375	–0.231, 0.981	0.230
2’FL	0.129	–0.982, 1.240	0.820	–0.136	–1.201, 0.928	0.803
3FL	–0.129	–0.407, 0.149	0.363	0.262	–0.067, 0.590	0.123
LNnT	–0.172	–0.347, 0.002	0.055	–0.203	–0.485, 0.079	0.164
3’SL	–0.099	–0.285, 0.086	0.295	–0.110	–0.632, 0.412	0.680
DFLac	0.085	–0.332, 0.501	0.690	0.683	0.060, 1.307	0.036
6’SL	0.151	–0.013, 0.315	0.072	0.224	–0.113, 0.561	0.198
LNT	–0.014	–0.190, 0.163	0.880	0.093	–0.218, 0.404	0.560
LNFP I	–0.117	–0.501, 0.267	0.552	0.171	–0.517, 0.858	0.628
LNFP II	–0.019	–0.231, 0.193	0.861	–0.117	–0.482, 0.248	0.531
LNFP III	0.018	–0.165, 0.200	0.848	0.183	–0.166, 0.531	0.308
LSTb	–0.125	–0.295, 0.045	0.151	–0.041	–0.417, 0.336	0.833
LSTc	–0.215	–0.416, -0.014	0.037	–0.074	–0.369, 0.220	0.623
DFLNT	–0.035	–0.244, 0.174	0.741	–0.151	–0.613, 0.312	0.526
LNH	–0.066	–0.247, 0.116	0.479	–0.014	–0.327, 0.299	0.931
DSLNT	–0.033	–0.199, 0.134	0.701	0.105	–0.232, 0.441	0.545
FLNH	–0.053	–0.235, 0.129	0.571	0.072	–0.224, 0.367	0.636
DFLNH	–0.008	–0.222, 0.206	0.943	0.050	–0.532, 0.632	0.866
FDSLNH	0.056	–0.152, 0.264	0.598	–0.033	–0.446, 0.380	0.878
DSLNH	–0.009	–0.170, 0.152	0.913	–0.007	–0.378, 0.365	0.972
Total HMO	–0.182	–0.812, 0.447	0.571	–0.570	–1.774, 0.634	0.357
HMO-bound Sia	0.019	–0.158, 0.196	0.833	0.126	–0.224, 0.477	0.483
HMO-bound Fuc	0.080	–0.646, 0.806	0.830	–0.117	–0.536, 0.302	0.587
2’FL/LNnT ratio	0.478	–0.069, 1.025	0.088	0.258	–0.448, 0.965	0.476

Estimates are average change in outcome *z*-score/1 SD change in Simpson diversity, or 1 SD change in log concentrations of individual HMOs, total HMO, fuc, and sia, or 1 SD change in the log-ratio of 2'FL and LNnT concentrations. Models were hierarchical mixed-effects linear models adjusted for maternal prepregnancy BMI, household income during pregnancy, infant sex, human milk feeding duration in completed weeks (up to 12 mo), any introduction to formula milk feeding by 12 mo, and the corresponding *z*-score at birth. All models include an interaction term between HMO and time and use an unstructured correlation structure and a random intercept for each participant.

2’FL, 2’-fucosyllactose; 3’SL, 3’-sialyllactose; 3FL, 3-fucosyllactose; 6’SL, 6’-sialyllactose; DFLac, difucosyllactose; DFLNH, difucosyllacto-N-hexaose; DFLNT, difucosyllacto-N-tetraose; DSLNH, disialyllacto-N-hexaose; DSLNT, disialyllacto-N-tetraose; FDSLNH, fucodisialyllacto-N-hexaose; FLNH, fucosyllacto-N-hexaose; Fuc, HMO-bound fucose; HMO, human milk oligosaccharide; LNFP, lacto-N-fucopentaose; LNH, lacto-N-hexaose; LNnT, lacto-N-neotetraose; LNT, lacto-N-tetraose; LSTb, sialyl-lacto-N-tetraose b; LSTc, sialyl-lacto-N-tetraose c; Sia, HMO-bound sialic acid.

Few of the associations between HMOs and anthropometric measures observed in the 6 wk to 6 mo of age models were evident in the models at 12 mo to 4 y of age. Exceptions included higher 2’FL still associated with greater head circumference *z*-score [1.05 SD (0.17, 1.93), *P* = 0.02] (Supplementary Figure 6) in children born to nonsecretor mothers. In the overall cohort, lower LNnT, higher 6’SL, and lower sialyl-lacto-N-tetraose c were associated with greater skinfold thickness *z*-scores in the 12 mo to 4 y models (Supplementary Figure 8).

Post hoc investigation of model assumptions for the study findings indicated that model assumptions were reasonable except for equal variance of residuals across participants, with the majority of models showing evidence of unequal variances (*P* < 0.05).

## Discussion

In this Australian population-derived cohort of children and their mothers, HMO concentrations in milk at 6 wk postpartum were tested for associations with growth in infancy. We observed associations between specific HMO concentrations and changes in head circumference *z*-score from birth and found evidence for potentially secretor status-specific associations between several other HMOs with weight and length *z*-scores and, to a lesser degree, weight-for-length, and sum of skinfold thickness *z*-scores. We replicated previous findings of associations between higher 2’FL and increased weight and length *z*-scores in children born to secretor mothers only, but this association was only evident at 6 mo of age, in contrast to previous findings supporting these relationships continuing to 5 y of age [12].

In this study, the direction of associations differed by HMO and anthropometric *z*-score, but generally, the largest association per SD difference in HMO concentration was seen for 2’FL, the most abundant HMO in secretor mother’s milk. Less compelling evidence was apparent linking HMO concentrations with weight-for-length or sum of skinfold thickness *z*-scores (2 measures of body composition in infancy), suggesting that the HMO concentration may more strongly associate with overall child size rather than body composition, at least for growth up to 6 mo of age. Such findings are consistent with previous evidence suggesting that HMO concentrations may modulate the effect of human milk feeding on child growth [[12], [13], [14]].

The head circumference has not been investigated by many prior studies. One smaller study (*n* = 50) reported no evidence for associations between 5 HMOs (2’FL, LNT, LNnT, 3’SL, and 6’SL) and infant head circumference up to 4 mo of age [27]. However, the associations we observed with a head circumference at 6 wk and 6 mo included 2’FL and 6’SL as well as other HMO measures not considered in the previous study (DSLNH and sia). Differences in findings may be related to *1*) different time points for head measurement, *2*) ethnicity of participants (Chinese ethnicity as opposed to the largely European Barwon Infant Study cohort) [17], or *3*) the previously documented widespread variation in milk HMO composition geographically [28].

In a previous study investigating change in weight and length *z*-scores from birth to 5 y of age (*n* = 802 infants, 87% born to secretor mothers) [12], several HMO measures (including 2’FL, LNnT, LSTb, fuc, and HMO diversity) were associated with weight and length in children born to secretor mothers only. We observed some of these same associations (2’FL, LSTb, fucose with weight, and 2’FL with length). There was weaker evidence of other previously reported associations, possibly because of the smaller cohort size and fewer time points in our study resulting in lower statistical power. Notwithstanding, we did observe the same direction and similar magnitude of associations up to 6 mo of age. A study investigating HMOs and excessive weight gain (defined as a weight-for-age *z*-score at 5 mo of at least 2, and at least 1 SD greater than weight-for-age *z*-score at birth) up to 9 mo of age in a smaller cohort (*n* = 28 infants, 82% born to secretor mothers, *n* = 11 in high weight gain group) [13] also found evidence for positive associations of 2’FL, fuc, and total HMO with higher weight velocity and fat-mass index and/or greater odds of being in the high weight gain group at 5 mo of age for infants born to secretor mothers, consistent with the direction of our findings with weight *z*-score.

There are less data exploring the relationships between HMO and adiposity measures. However, in a small study (*n* = 25 infants, 18 secretor mothers), there were cross-sectional associations of higher HMO diversity at 1 mo of age with lower infant fat mass, higher LNFP I at both 1 mo and 6 mo with lower weight, and higher DSLNT and LNFP II at 6 mo with greater fat mass [14]. In addition, although direct measures of fat mass were not available in our infants at 1 mo, we observed evidence of associations between higher HMO diversity and lower change in both sums of skinfold thickness and weight *z*-scores. We also observed evidence of associations between LNFP I and higher length *z*-score (up to 6 mo of age, in children born to nonsecretor mothers only) and greater head circumference (in 12 mo to 4 y of age model, overall cohort), but did not observe any clear evidence for associations of LNFP II or DSLNT with anthropometry in either secretor group in this study.

Although this and previous studies provide evidence for HMOs potentially playing a role in mediating the effect of human milk on child anthropometry, the causal mechanisms are not well established. HMOs are prebiotic compounds that influence the infant’s gastrointestinal microbiome in early life [29]. As evidence suggests that early-life gastrointestinal microbiomes can have long-lasting effects on the growing risk of obesity [30], it is plausible that even a short period of HMO exposure early in life could have long-term effects on offspring anthropometry through shaping the gastrointestinal microbiome. However, we did not observe clear evidence for persistent associations of specific HMOs on anthropometry to 4 y of age. Additionally, HMOs may have direct, microbiota-independent effects on host metabolism, immune responses, and more [[31], [32], [33]], but these are difficult to assess in the absence of direct microbiome measures.

Human milk is considered the ideal source of nutrition for infants, but a substantial proportion of infants receive formula to varying extents. The mounting evidence of the beneficial role of HMOs suggests a potential benefit to including these in commercially available formulas. Indeed, several infant formula products are already supplemented with some HMOs, including 2’FL and LNnT [34]. However, further studies are needed to understand better the potential effects of HMOs – either alone or in combination – on a deep mechanistic level to unlock the full potential of HMOs in supporting infant health and development.

This study is the first Australian cohort to consider the relationship between HMO concentrations in milk and anthropometry in early life and the first of its size to consider these anthropometric measures up to 4 y of age to investigate persistent effects beyond lactation. Limitations include the modest sample size and single time point of milk sampling, which precludes analyzing changes in HMO composition over lactation. In addition, data on supplementary formula feeding were not collected for infants who were not exclusively human milk-fed, so we were unable to assess how the relative amount of human milk to formula milk an infant received might modify these associations. The lack of detailed data on infant diet means we are unable to rule out the influence of later feeding patterns on anthropometric measures up to 4 y. It is important to consider these findings with caution in the context of the large number of models investigated in this study, as model results were not adjusted for multiple comparisons. Some findings may be because of chance, so replication will be important to strengthen confidence, particularly for novel observed associations.

As previously reported, we found evidence for specific HMOs at 6 wk postpartum associating with different anthropometric measures in infancy and childhood up to 4 y of age, including associations of 2’FL and other HMOs with change in weight and length *z*-scores. Further, we identified associations between several HMOs and other growth and body composition measures, including head circumference. Relationships between HMOs and growth measures differed according to the age of the offspring. Although we were not able to assess causality, our findings suggest that a constellation of short- and longer-term effects on offspring growth could be conferred by HMOs and that other factors are likely to be important in an age-dependent manner. Inter-individual variation in HMO concentrations and the prevalence of secretor status-specific associations with growth may contribute to the inconsistent epidemiological findings linking human milk feeding with offspring growth and obesity risk. Understanding the causal mechanisms through which HMOs influence child growth and body composition may identify opportunities for supplementation or intervention to promote healthy child growth and reduce the risk of metabolic diseases later in life.

Members of the Barwon Infant Study Investigator Group are as follows– Peter Vuillermin, Anne-Louise Ponsonby, John Carlin, Mimi LK Tang, Fiona Collier, Amy Loughman, Toby Mansell, Lawrence Gray, Martin O’Hely, Richard Saffery, Sarath Ranganathan, David Burgner, Peter Sly. We thank Terry Dwyer and Katie Allen for their past work as foundation investigators.

## Author contribution

The authors’ responsibilities were as follows– TM, AF, MOH, A-LP, PV, DB, RS, and LB: conceptualized and developed this study and drafted the manuscript; TM: led the data analysis; MOH: was a major contributor to the analysis; MC: performed human milk oligosaccharides measurements; AF, A-LP, PV, DB, RS, and LB: were major contributors to the interpretation of data; All authors provided critical expert advice and a critical review and all authors: read and approved the final manuscript.

## Conflict of interest

LB is a co-inventor on patent applications related to using HMOs to prevent NEC and other inflammatory diseases. TM is supported by an MCRI ECR Fellowship. This work was also supported by NHMRC Senior Research Fellowships (1064629 to DB; 1045161 to RS), an NHMRC Investigator grant (1175744 to DB), and a Eunice Kennedy Shriver National Institute of Child Health and Human Development grant (R21 HD105186 to LB). All other authors report no conflicts of interest.

## Data Availability

Data described in the manuscript, code book, and analytic code will be made available upon request, pending application and approval from Barwon Infant Study data custodians.

## Funding

The establishment work and infrastructure for the Barwon Infant Study were provided by the Murdoch Children’s Research Institute, Deakin University, and Barwon Health. Subsequent funding was secured from the National Health and Medical Research Council of Australia, The Jack Brockhoff Foundation, the Scobie Trust, the Shane O’Brien Memorial Asthma Foundation, the Our Women’s Our Children’s Fund-Raising Committee Barwon Health, The Shepherd Foundation, the Rotary Club of Geelong, the Ilhan Food Allergy Foundation, GMHBA Limited and the Percy Baxter Charitable Trust, Perpetual Trustees, and the Minderoo Foundation. In addition, in-kind support was provided by the Cotton On Foundation and CreativeForce. Research at Murdoch Children’s Research Institute is supported by the Victorian Government’s Operational Infrastructure Support Program. The funding bodies were not involved in the study design; collection, analysis, and interpretation of the data; writing and preparation of the manuscript; or decision to submit the manuscript for publication.

## Appendix ASupplementary data

Supplementary Figures 1-8, Supplementary Tables 1 and 3 Supplementary Figures 1-8, Supplementary Tables 1 and 3
