# Supplementary material for: Age-dependent associations of human milk oligosaccharides with body size and composition up to 4 years of age
Source: Am J Clin Nutr. 2023 Feb 21;117(5):930–45. doi: 10.1016/j.ajcnut.2023.02.016 (PMC10447468; doi:10.1016/j.ajcnut.2023.02.016)
Supplement: Supplementary Figures 1-8, Supplementary Tables 1 and 3 [file mmc1.docx]

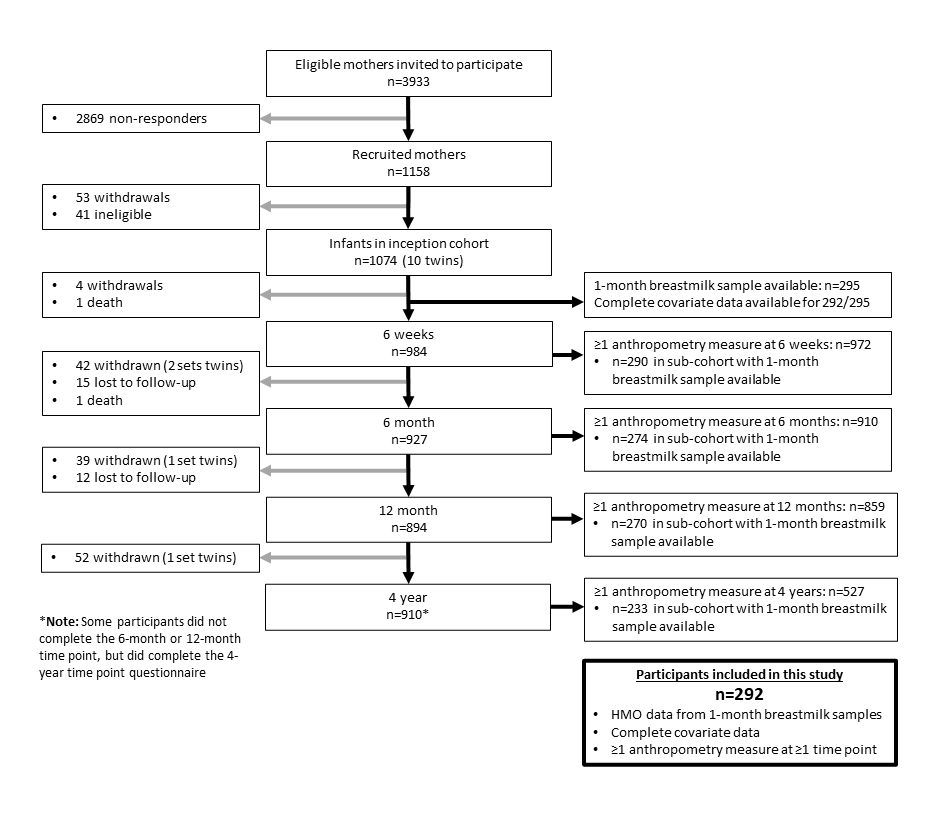


**Supplementary Figure 1.** Flowchart of Barwon Infant Study participants included this study (bolded box). Included participants with HMO data from 1-month breastmilk samples, at least 1 anthropometry measure from at least 1 time point, and complete covariate data.


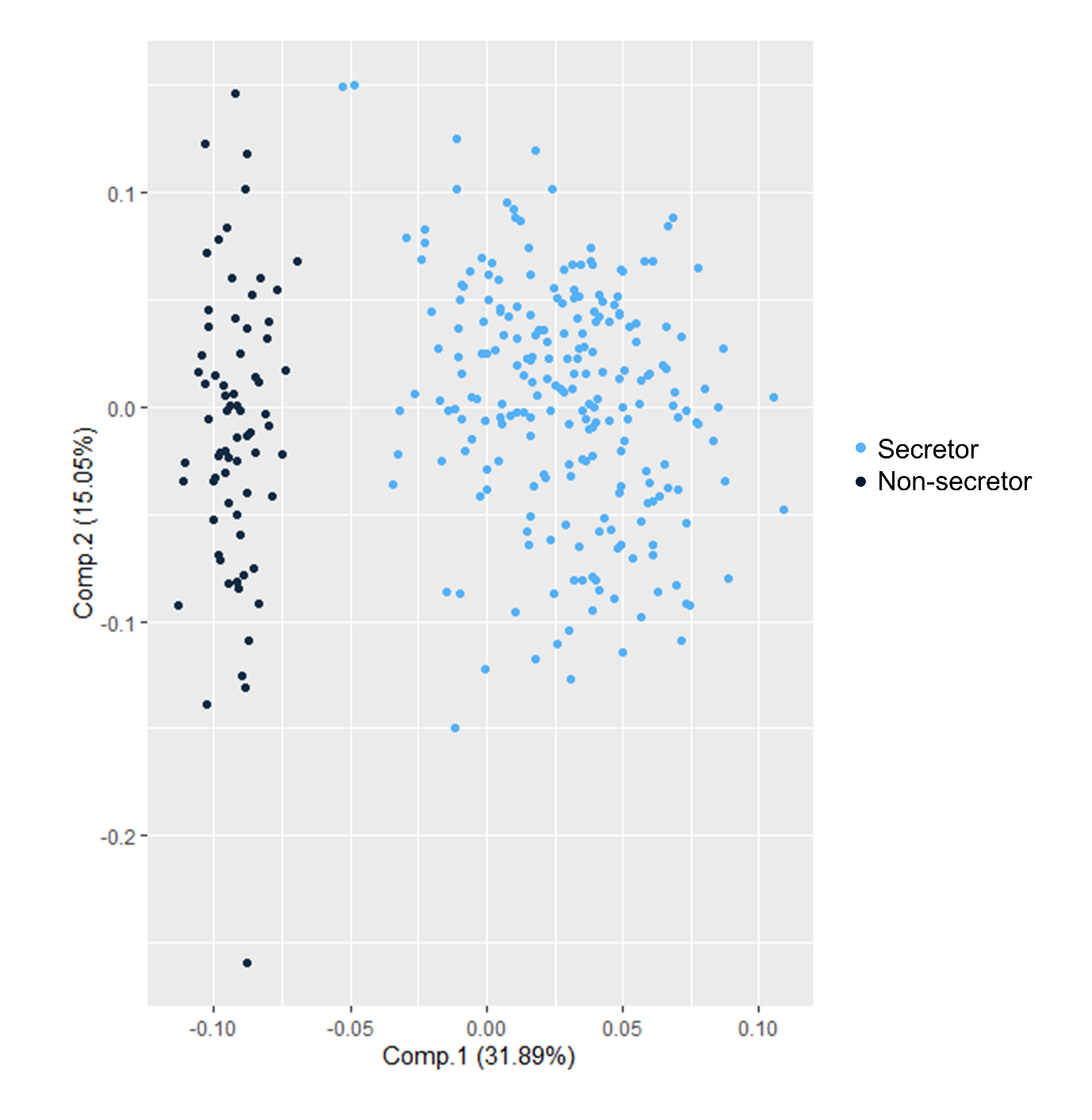


**Supplementary Figure 2.** Principal component analysis plot of variation in HMO composition for each breast milk sample (principal component 1 on x-axis, principal component 2 on y-axis), coloured by secretor status (secretor: blue; non-secretor: black). Percentage of HMO composition variation explained by each principal component is displayed in brackets.


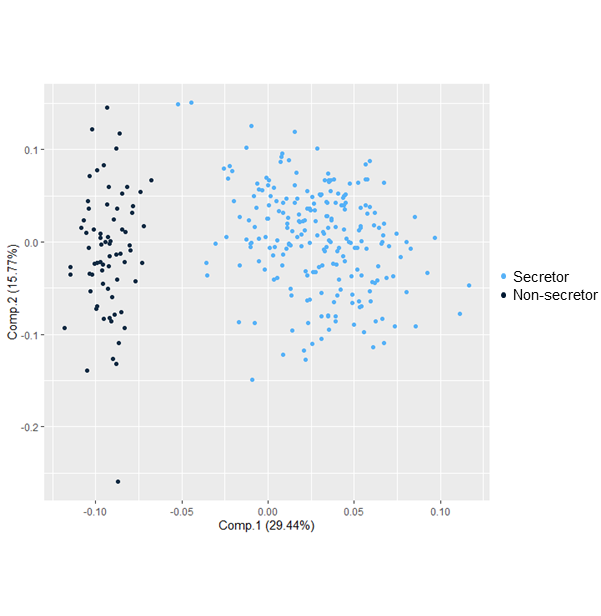


**Supplementary Figure 3.** Principal component analysis plot of variation in HMO composition (excluding 2’FL) for each breast milk sample (principal component 1 on x-axis, principal component 2 on y-axis), coloured by secretor status (secretor: blue; non-secretor: black). Percentage of HMO composition variation explained by each principal component is displayed in brackets.


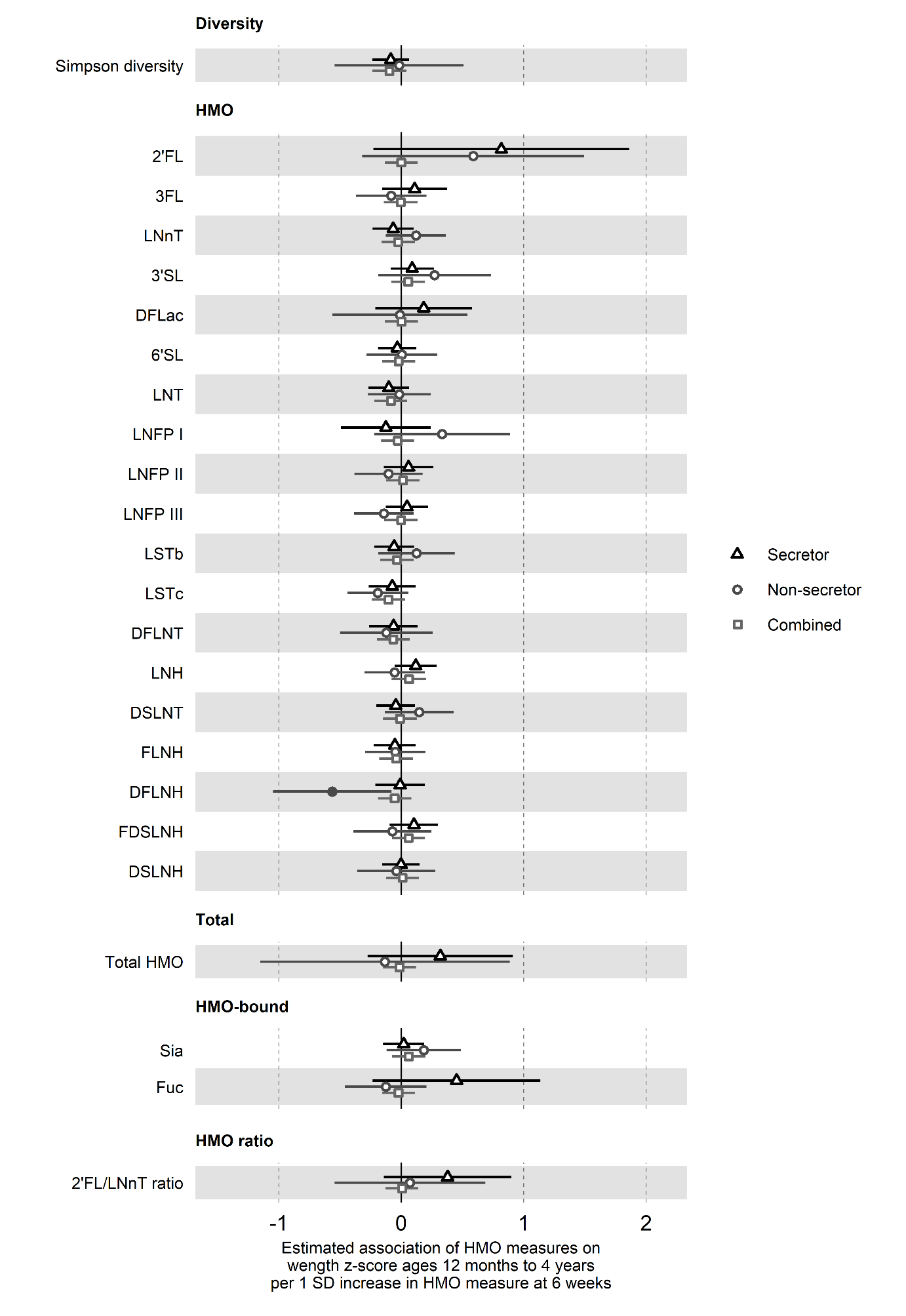


**Supplementary Figure 4.** Estimated association of 1 SD increase in HMO measures on weight z-score at 12 months and 4 years of age.

Forest plots of the estimated difference in weight z-score (SD units) at 12 months and 4 years of age per 1 SD increase in HMO measure, from hierarchical mixed-effects linear models adjusted for weight z-score at birth and potential confounders. Secretor status-stratified models are depicted with triangles (secretor only, n=201) and circles (non-secretor only, n=66), and combined models with the overall cohort are squares (n=267). All HMO measures other than diversity were log-transformed prior to analysis. Error bars are 95% confidence intervals. Closed points represent p<0.05. HMOs are the log-concentrations of the 19 species of HMO measured. Diversity is the Shannon diversity of these HMOs. Total is the total concentration of the 19 HMOs. HMO-bound is the log-concentrations of HMO-bound sialic acid and fucose. 2’FL: 2’-fucosyllactose. 3FL: 3-fucosyllactose. 3’SL: 3’-sialyllactose. 6’SL: 6’-sialyllactose. DFLac: difucosyllactose. DFLNH: difucosyllacto-N-hexaose. DFLNT: difucosyllacto-N-tetraose. DSLNH: disialyllacto-N-hexaose. DSLNT: disialyllacto-N-tetraose. FDSLNH: fucodisialyllacto-N-hexaose. FLNH: fucosyllacto-N-hexaose. Fuc: HMO-bound fucose. HMO: human milk oligosaccharide. LNFP: lacto-N-fucopentaose. LNH: lacto-N-hexaose. LNnT: lacto-N-neotetraose. LNT: lacto-N-tetraose. LSTb: sialyl-lacto-N-tetraose b. LSTc: sialyl-lacto-N-tetraose c. Sia: HMO-bound sialic acid.


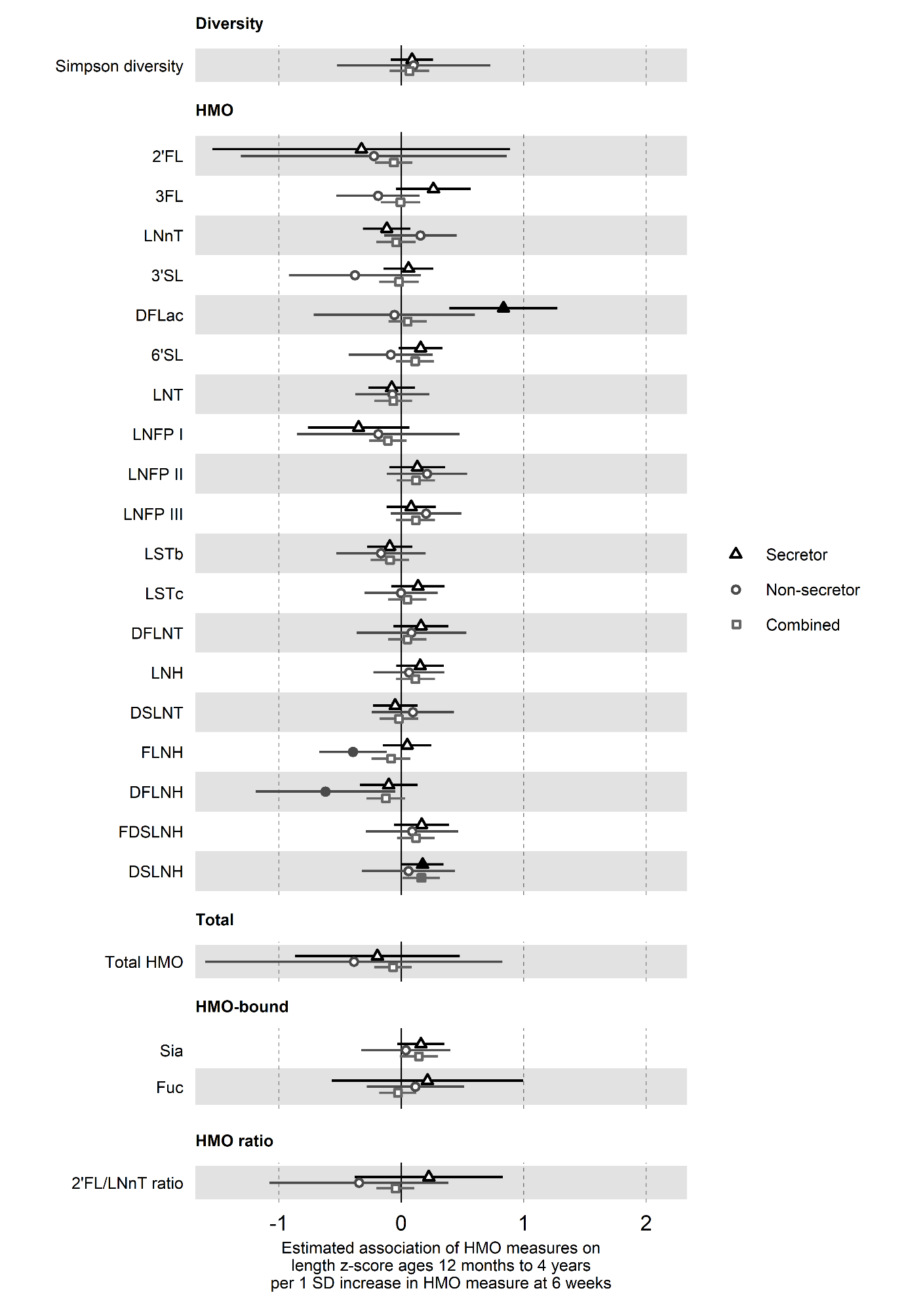


**Supplementary Figure 5.** Estimated association of 1 SD increase in HMO measures on length z-score at 12 months and 4 years of age.

Forest plots of the estimated difference in length z-score (SD units) at 12 months and 4 years of age per 1 SD increase in HMO measure, from hierarchical mixed-effects linear models adjusted for length z-score at birth and potential confounders. Secretor status-stratified models are depicted with triangles (secretor only, n=199) and circles (non-secretor only, n=66), and combined models with the overall cohort are squares (n=265). All HMO measures other than diversity were log-transformed prior to analysis. Error bars are 95% confidence intervals. Closed points represent p<0.05. HMOs are the log-concentrations of the 19 species of HMO measured. Diversity is the Shannon diversity of these HMOs. Total is the total concentration of the 19 HMOs. HMO-bound is the log-concentrations of HMO-bound sialic acid and fucose. 2’FL: 2’-fucosyllactose. 3FL: 3-fucosyllactose. 3’SL: 3’-sialyllactose. 6’SL: 6’-sialyllactose. DFLac: difucosyllactose. DFLNH: difucosyllacto-N-hexaose. DFLNT: difucosyllacto-N-tetraose. DSLNH: disialyllacto-N-hexaose. DSLNT: disialyllacto-N-tetraose. FDSLNH: fucodisialyllacto-N-hexaose. FLNH: fucosyllacto-N-hexaose. Fuc: HMO-bound fucose. HMO: human milk oligosaccharide. LNFP: lacto-N-fucopentaose. LNH: lacto-N-hexaose. LNnT: lacto-N-neotetraose. LNT: lacto-N-tetraose. LSTb: sialyl-lacto-N-tetraose b. LSTc: sialyl-lacto-N-tetraose c. Sia: HMO-bound sialic acid.


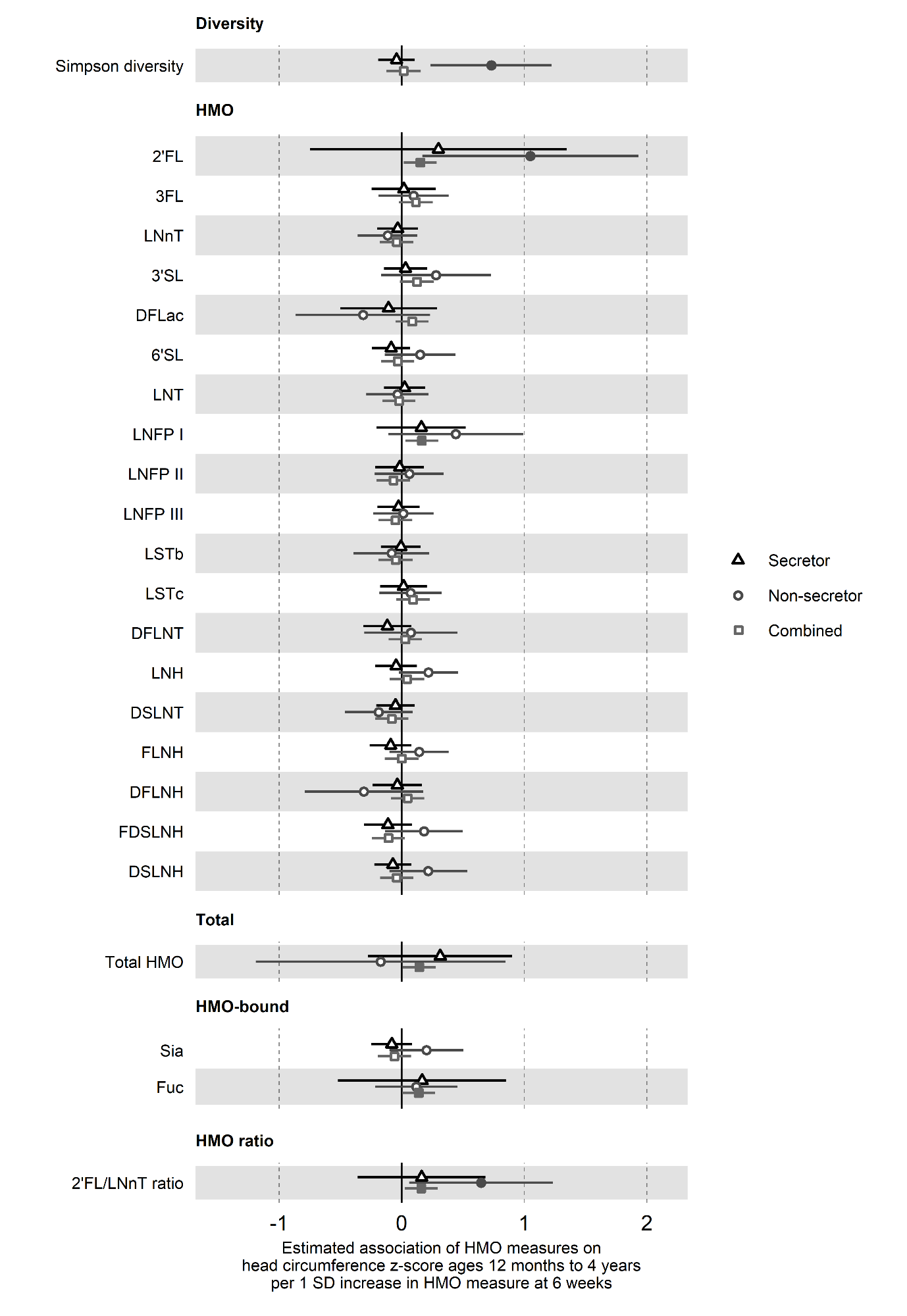


**Supplementary Figure S6.** Estimated association of 1 SD increase in HMO measures on head circumference z-score at 12 months and 4 years of age.

Forest plots of the estimated difference in head circumference z-score (SD units) at 12 months and 4 years of age per 1 SD increase in HMO measure, from hierarchical mixed-effects linear models adjusted for head circumference z-score at birth and potential confounders. Secretor status-stratified models are depicted with triangles (secretor only, n=210) and circles (non-secretor only, n=69), and combined models with the overall cohort are squares (n=279). All HMO measures other than diversity were log-transformed prior to analysis. Error bars are 95% confidence intervals. Closed points represent p<0.05. HMOs are the log-concentrations of the 19 species of HMO measured. Diversity is the Shannon diversity of these HMOs. Total is the total concentration of the 19 HMOs. HMO-bound is the log-concentrations of HMO-bound sialic acid and fucose. 2’FL: 2’-fucosyllactose. 3FL: 3-fucosyllactose. 3’SL: 3’-sialyllactose. 6’SL: 6’-sialyllactose. DFLac: difucosyllactose. DFLNH: difucosyllacto-N-hexaose. DFLNT: difucosyllacto-N-tetraose. DSLNH: disialyllacto-N-hexaose. DSLNT: disialyllacto-N-tetraose. FDSLNH: fucodisialyllacto-N-hexaose. FLNH: fucosyllacto-N-hexaose. Fuc: HMO-bound fucose. HMO: human milk oligosaccharide. LNFP: lacto-N-fucopentaose. LNH: lacto-N-hexaose. LNnT: lacto-N-neotetraose. LNT: lacto-N-tetraose. LSTb: sialyl-lacto-N-tetraose b. LSTc: sialyl-lacto-N-tetraose c. Sia: HMO-bound sialic acid.
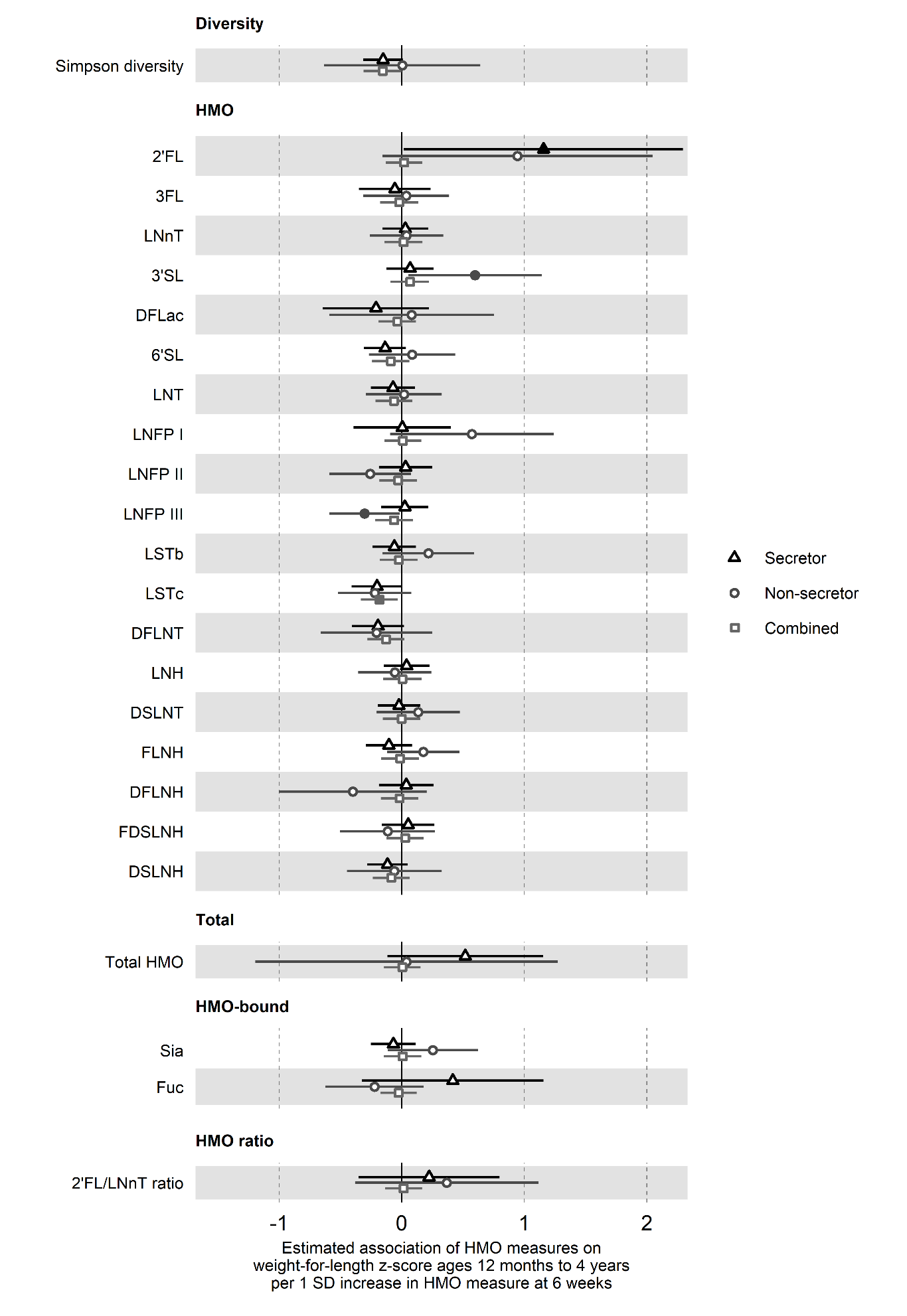


**Supplementary Figure S7.** Estimated association of 1 SD increase in HMO measures on weight-for-length z-score at 12 months and 4 years of age.

Forest plots of the estimated difference in weight-for-length z-score (SD units) at 12 months and 4 years of age per 1 SD increase in HMO measure, from hierarchical mixed-effects linear models adjusted for weight-for-length z-score at birth and potential confounders. Secretor status-stratified models are depicted with triangles (secretor only, n=199) and circles (non-secretor only, n=66), and combined models with the overall cohort are squares (n=265). All HMO measures other than diversity were log-transformed prior to analysis. Error bars are 95% confidence intervals. Closed points represent p<0.05. HMOs are the log-concentrations of the 19 species of HMO measured. Diversity is the Shannon diversity of these HMOs. Total is the total concentration of the 19 HMOs. HMO-bound is the log-concentrations of HMO-bound sialic acid and fucose. 2’FL: 2’-fucosyllactose. 3FL: 3-fucosyllactose. 3’SL: 3’-sialyllactose. 6’SL: 6’-sialyllactose. DFLac: difucosyllactose. DFLNH: difucosyllacto-N-hexaose. DFLNT: difucosyllacto-N-tetraose. DSLNH: disialyllacto-N-hexaose. DSLNT: disialyllacto-N-tetraose. FDSLNH: fucodisialyllacto-N-hexaose. FLNH: fucosyllacto-N-hexaose. Fuc: HMO-bound fucose. HMO: human milk oligosaccharide. LNFP: lacto-N-fucopentaose. LNH: lacto-N-hexaose. LNnT: lacto-N-neotetraose. LNT: lacto-N-tetraose. LSTb: sialyl-lacto-N-tetraose b. LSTc: sialyl-lacto-N-tetraose c. Sia: HMO-bound sialic acid.


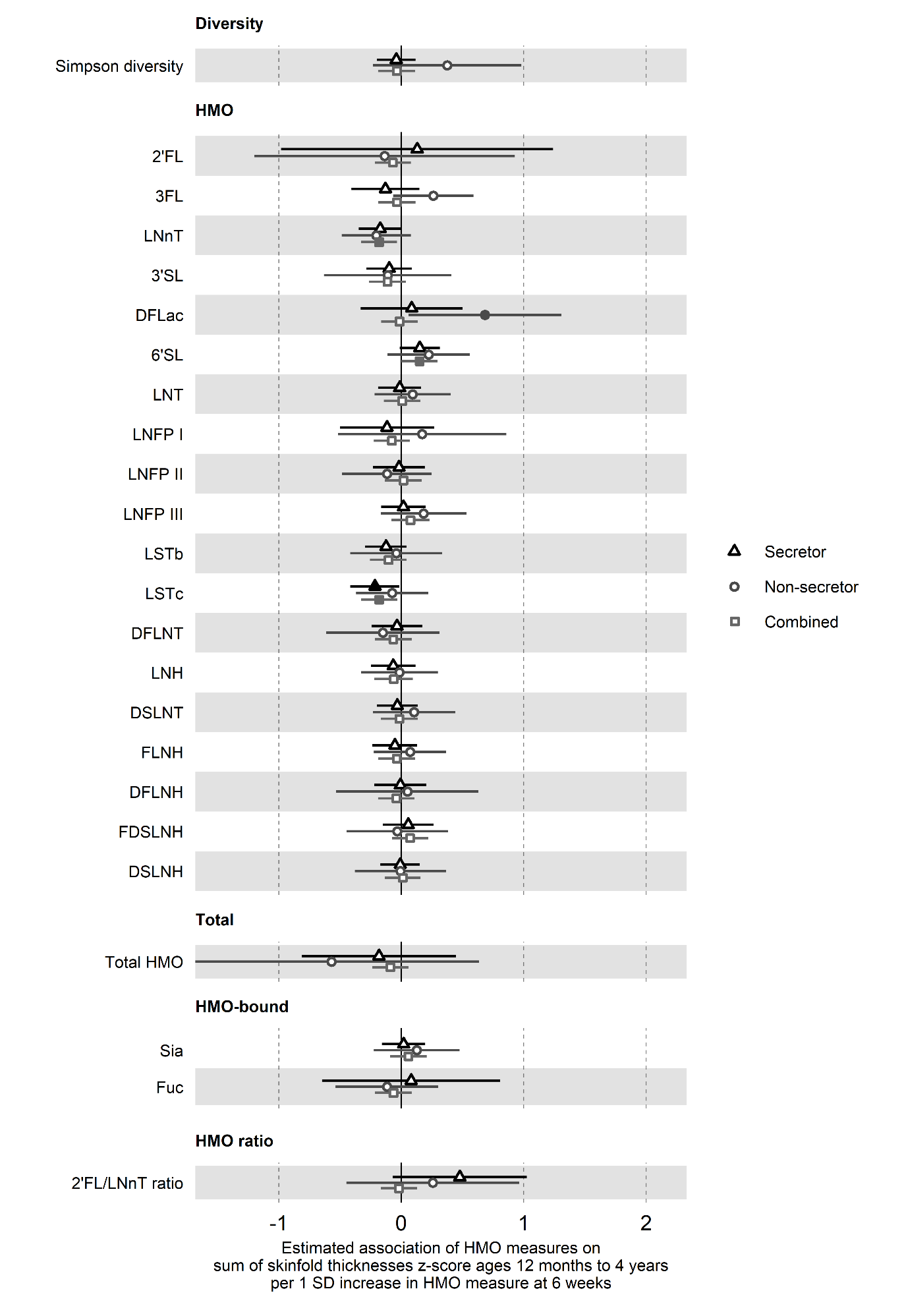


**Supplementary Figure S8.** Estimated association of 1 SD increase in HMO measures on sum of skinfold thickness z-score at 12 months and 4 years of age.

Forest plots of the estimated difference in sum of skinfold thickness z-score (SD units) at 12 months and 4 years of age per 1 SD increase in HMO measure, from hierarchical mixed-effects linear models adjusted for sum of skinfold thickness z-score at birth and potential confounders. Secretor status-stratified models are depicted with triangles (secretor only, n=208) and circles (non-secretor only, n=69), and combined models with the overall cohort are squares (n=277). All HMO measures other than diversity were log-transformed prior to analysis. Error bars are 95% confidence intervals. Closed points represent p<0.05. HMOs are the log-concentrations of the 19 species of HMO measured. Diversity is the Shannon diversity of these HMOs. Total is the total concentration of the 19 HMOs. HMO-bound is the log-concentrations of HMO-bound sialic acid and fucose. 2’FL: 2’-fucosyllactose. 3FL: 3-fucosyllactose. 3’SL: 3’-sialyllactose. 6’SL: 6’-sialyllactose. DFLac: difucosyllactose. DFLNH: difucosyllacto-N-hexaose. DFLNT: difucosyllacto-N-tetraose. DSLNH: disialyllacto-N-hexaose. DSLNT: disialyllacto-N-tetraose. FDSLNH: fucodisialyllacto-N-hexaose. FLNH: fucosyllacto-N-hexaose. Fuc: HMO-bound fucose. HMO: human milk oligosaccharide. LNFP: lacto-N-fucopentaose. LNH: lacto-N-hexaose. LNnT: lacto-N-neotetraose. LNT: lacto-N-tetraose. LSTb: sialyl-lacto-N-tetraose b. LSTc: sialyl-lacto-N-tetraose c. Sia: HMO-bound sialic acid.

**Supplementary Table 1.** Distribution of milk HMO measures, by secretor status.

| **Measure** | **Combined** | **Non-secretor** | **Secretor** |
| --- | --- | --- | --- |
|  | N = 292 | N = 71 | N = 221 |
| Diversity | 5.8 [4.8-6.9] | 5.7 [5.2-6.1] | 6.0 [4.7-7.2] |
| 2’FL (nmol/L) | 4,476.4 [1,671.9-6,328.4] | 14.3 [8.8-24.6] | 5,473.6 [3,991.0-6,900.4] |
| 3FL (nmol/L) | 212.2 [140.3-297.5] | 90.9 [58.8-131.1] | 249.1 [189.0-330.4] |
| LNnT (nmol/L) | 694.1 [459.2-960.8] | 644.2 [363.9-938.3] | 706.1 [487.5-978.0] |
| 3’SL (nmol/L) | 477.4 [311.8-697.4] | 288.1 [236.8-330.9] | 560.8 [399.7-747.7] |
| DFLac (nmol/L) | 316.6 [82.3-442.4] | 22.0 [17.4-33.5] | 377.8 [286.7-488.0] |
| 6’SL (nmol/L) | 515.6 [327.1-751.3] | 472.9 [324.9-696.9] | 518.1 [327.3-771.4] |
| LNT (nmol/L) | 1,408.8 [1,045.1-1,814.6] | 1,570.2 [1,139.9-2,099.3] | 1,362.3 [1,000.2-1,676.3] |
| LNFP I (nmol/L) | 1,051.2 [401.6-1,616.7] | 114.8 [81.7-151.2] | 1,277.2 [884.9-1,841.5] |
| LNFP II (nmol/L) | 1,569.8 [1,120.6-2,343.8] | 2,952.3 [2,544.3-3,157.1] | 1,399.9 [1,005.8-1,786.5] |
| LNFP III (nmol/L) | 74.7 [55.2-96.1] | 87.7 [68.8-106.6] | 70.3 [52.2-90.5] |
| LSTb (nmol/L) | 137.1 [104.6-174.4] | 161.4 [133.9-195.3] | 126.7 [98.0-167.4] |
| LSTc (nmol/L) | 174.7 [114.9-236.3] | 110.8 [69.6-150.5] | 189.3 [145.0-263.5] |
| DFLNT (nmol/L) | 1,661.4 [761.8-2,075.1] | 557.7 [421.1-722.4] | 1,888.0 [1,524.7-2,185.9] |
| LNH (nmol/L) | 94.3 [67.9-125.3] | 90.0 [59.3-120.4] | 96.3 [69.9-126.9] |
| DSLNT (nmol/L) | 473.5 [376.1-606.7] | 462.5 [375.8-612.6] | 496.8 [378.0-606.1] |
| FLNH (nmol/L) | 104.3 [61.5-170.4] | 77.6 [39.5-116.4] | 120.2 [70.3-180.6] |
| DFLNH (nmol/L) | 74.2 [30.8-143.7] | 22.7 [17.3-30.1] | 108.5 [59.7-178.1] |
| FDSLNH (nmol/L) | 283.7 [168.5-458.9] | 667.9 [484.0-886.4] | 228.6 [134.8-342.9] |
| DSLNH (nmol/L) | 170.8 [129.9-236.5] | 177.7 [138.2-239.2] | 169.6 [126.9-231.9] |
| Total HMO (nmol/L) | 15,490.2 [13,449.2-16,441.4] | 8,981.4 [8,683.9-9,363.3] | 15,971.9 [15,229.0-16,730.2] |
| HMO-bound Sia (nmol/L) | 3,495.3 [3,037.5-4,067.0] | 4,047.1 [3,471.3-4,359.3] | 3,357.2 [2,958.4-3,852.7] |
| HMO-bound Fuc (nmol/L) | 13,186.9 [9,888.6-14,469.8] | 5,458.2 [4,909.9-5,910.4] | 13,928.0 [12,948.6-14,828.5] |
| 2FL/LNnT ratio | 5.8 [1.6-11.6] | 0.0 [0.0-0.1] | 8.1 [4.5-14.1] |

HMO variables are reported as median [IQR].

**Supplementary Table 3.** Association between secretor status and anthropometry z-scores (infants born to secretor mothers compared to infants born to non-secretor mothers).

|  | **6 weeks to 6 months** | | | **12 months to 4 years** | | |
| --- | --- | --- | --- | --- | --- | --- |
| **Measure** | **Estimate (β)** | **95% confidence interval** | **P-value** | **Estimate (β)** | **95% confidence interval** | **P-value** |
| Height z-score | -0.034 | (-0.332, 0.264) | 0.825 | -0.126 | (-0.485, 0.233) | 0.493 |
| Weight z-score | -0.021 | (-0.251, 0.21) | 0.861 | -0.078 | (-0.390, 0.234) | 0.624 |
| Weight-for-length z-score | -0.012 | (-0.335, 0.311) | 0.941 | -0.049 | (-0.399, 0.301) | 0.785 |
| Head circumference z-score | -0.016 | (-0.326, 0.295) | 0.922 | 0.300 | (-0.014, 0.613) | 0.062 |
| Sum of skinfold thicknesses z-score | 0.160 | (-0.146, 0.465) | 0.306 | -0.182 | (-0.526, 0.162) | 0.301 |

Estimates are average difference in outcome z-score in infants born to secretor mothers (secretor group) compared to infants born to non-secretor mothers (non-secretor group).

Models were hierarchical mixed-effects linear models adjusted for maternal pre-pregnancy BMI, household income during pregnancy, infant sex, breastfeeding duration in completed weeks (up to 6 months), and the corresponding z-score at birth.

All models include an interaction term between secretor status and time, and use an unstructured correlation structure and a random intercept for each participant.
